# Immortalized hepatocyte-like cells: A competent hepatocyte model for studying clinical HCV isolate infection

**DOI:** 10.1371/journal.pone.0303265

**Published:** 2024-05-13

**Authors:** Yongyut Pewkliang, Piyanoot Thongsri, Phichaya Suthivanich, Nipa Thongbaiphet, Jiraporn Keatkla, Ekawat Pasomsub, Usanarat Anurathapan, Suparerk Borwornpinyo, Adisak Wongkajornsilp, Suradej Hongeng, Khanit Sa-ngiamsuntorn

**Affiliations:** 1 Faculty of Medicine Ramathibodi Hospital, Program in Translational Medicine, Mahidol University, Rama VI Road, Rajathevi, Bangkok, Thailand; 2 Faculty of Science, Excellent Center for Drug Discovery, Mahidol University, Rama VI Road, Rajathevi, Bangkok, Thailand; 3 Faculty of Medicine Ramathibodi Hospital, Department of Pathology, Virology Laboratory, Mahidol University, Rajathevi, Bangkok, Thailand; 4 Faculty of Medicine Ramathibodi Hospital, Department of Pediatrics, Mahidol University, Rajathevi, Bangkok, Thailand; 5 Faculty of Science, Department of Biotechnology, Mahidol University, Rajathevi, Bangkok, Thailand; 6 Faculty of Medicine Siriraj Hospital, Department of Pharmacology, Mahidol University, Bangkok, Thailand; 7 Faculty of Pharmacy, Department of Biochemistry, Mahidol University, Rajathevi, Bangkok, Thailand; National Institute of Infectious Diseases: Kokuritsu Kansensho Kenkyujo, JAPAN

## Abstract

More than 58 million individuals worldwide are inflicted with chronic HCV. The disease carries a high risk of end stage liver disease, i.e., cirrhosis and hepatocellular carcinoma. Although direct-acting antiviral agents (DAAs) have revolutionized therapy, the emergence of drug-resistant strains has become a growing concern. Conventional cellular models, Huh7 and its derivatives were very permissive to only HCVcc (JFH-1), but not HCV clinical isolates. The lack of suitable host cells had hindered comprehensive research on patient-derived HCV. Here, we established a novel hepatocyte model for HCV culture to host clinically pan-genotype HCV strains. The immortalized hepatocyte-like cell line (imHC) derived from human mesenchymal stem cell carries HCV receptors and essential host factors. The imHC outperformed Huh7 as a host for HCV (JFH-1) and sustained the entire HCV life cycle of pan-genotypic clinical isolates. We analyzed the alteration of host markers (i.e., hepatic markers, cellular innate immune response, and cell apoptosis) in response to HCV infection. The imHC model uncovered the underlying mechanisms governing the action of IFN-α and the activation of sofosbuvir. The insights from HCV-cell culture model hold promise for understanding disease pathogenesis and novel anti-HCV development.

## Introduction

Hepatitis C virus (HCV) is a hepatotropic member of *Hepacivirus* and *Flaviviridae* family [1]. Worldwide, there were over 58 million reported cases of this pathogenic viral infection, with approximately 1.5 million new infections occurring each year and approximately 290,000 deaths in 2019 [2]. Chronic HCV infection could lead to chronic hepatitis, cirrhosis, and hepatocellular carcinoma (HCC) [3]. HCV-induced HCC has an annual incidence of 15,000 cases in USA [4]. In Thailand, between 760,000 and 790,000 cases have been reported, with approximately half of them being in chronic condition [5], highlighting its status as a critical public health concern [6]. HCV genome is a positive-stranded RNA **~**9.6 kb, encoding a core protein, two envelope glycoproteins (E1 and E2), and several non-structural proteins (NS2, NS3, NS4A, NS4B, NS5A and NS5B) [7,8]. Several cell culture systems were developed to investigate each step of viral life cycle, including HCV subgenomic replicon systems, HCV pseudoparticles (HCVpp) and cell culture-derived HCV (HCVcc) [9]. Particularly, the production of infectious HCV virions is required for dissecting viral biology and their response to antivirals. Only the JFH-1 strain of HCVcc (genotype 2a) could enter entire HCV life cycle in cultured hepatocyte models [10].

To examine the resistance of pan-viral genotypes to antivirals, it is necessary to utilize patient-derived HCV isolates, as the global incidence of HCV resistance has been steadily rising [11]. HCV research had been heavily relying on HCVcc (JFH-1) and its limited spectra of host cells (Huh7 and its derivatives, Huh7.5 and Huh7.5.1) [12–14]. However, these host cells carry unlimited proliferation, lack crucial hepatic markers (i.e., CYP450s and phase II and III drug metabolizing enzymes), and cannot host clinically isolated HCV. Thus, these cells might not adequately represent a natural host for HCV and are not suitable for evaluating anti-HCV drug activation and metabolism [15,16]. Primary human hepatocytes (PHHs) are considered a gold standard model for the study of hepatotropic pathogens [17,18]. Nevertheless, the shortcomings of PHHs, such as limited proliferation, limited donors, batch-to-batch variation, and the gradual decline of hepatic functions during culture, have rendered them unsuitable for experimental purposes [19,20]. Recently, induced pluripotent stem cell-derived hepatocyte-like cells (iHLCs) have been proposed as an alternative to PHHs [21] and used as cellular hosts for a full range of HCV that covered the clinical isolates [22–25]. Nevertheless, iHLCs exhibited some drawbacks: requiring extensive induction / characterization before use, immature hepatic functions, and limited life span [26,27].

To overcome the PHHs limitations, cancer cell-derived human hepatocyte cell, HLCZ01 was established to study the entire HCV life cycle from clinical isolates [28]. No explanation was provided for the inability of several hepatocyte models to host the replication of clinical HCV isolates. Huh7 and its derivatives (Huh7.5 and Huh7.5.1) were known for their robust ability to sustain high levels of HCVcc replication [14]. It was proposed that most cultured cells lack an essential factor, required for replication of clinical isolates [29]. The expression of host factor, *SEC14L2* (SEC14-like protein 2) enabled replication of pan HCV genotypes in hepatoma cell lines [29]. SEC14L2-expressing Huh-7.5 cells also supported HCV replication from patient sera, paving the way for the *in vitro* replication systems for all HCV isolates [29,30].

To substitute PHHs and iHLCs for *in vitro* study, a continuous non-cancerous hepatocyte cell line maintaining hepatic functions was required. The immortalized hepatocyte-like cell line (imHC) derived from human bone marrow mesenchymal stem cells (hMSCs) [16] exhibited essential hepatocyte phenotypes; ALB, AFP, HNF-4ɑ, G-6-Pase, TAT, urea metabolism, glycogen production, and all major CYP450s. Additionally, imHC could host human malarial parasite (*Plasmodium vivax*) [31], HBV [32] and dengue virus [33]. imHC highly expressed HCV-associated cell receptors (Claudin-1, Occludin, CD81, SR-BI, EphA2, EGFR, and LDLR) [34–36] and cell host factors (ApoB, ApoE, *miR-122* and SEC14L2) [29,37–40] that should support HCV replication. imHC could host pan-genotype HCV infection from clinical isolates and allowed full life cycle of HCV by producing complete HCV virions that could infect naïve imHC. Infected imHC also responded to treatments with interferon, ribavirin or sofosbuvir. Thus, imHC could serve as a model for drug sensitivity study in clinical isolated HCVs.

## Materials and methods

### Hepatocyte culture

HepG2 was obtained from the American Type Culture Collection (ATCC, HB-8065). Huh7 was obtained from the Japanese Collection of Research Bioresources (JCRB, JCRB0403). imHC was developed as previously described [41]. Briefly, imHC was prepared from hMSCs, immortalized with human telomerase transcriptase (*hTERT*) and *Bmi-1* via lentiviral vectors [42]. Subsequently, the immortalized hMSCs underwent cloning via serial dilution to achieve single cell per well. At least 6–8 clones were expanded and screened for the highest expression of *Bmi-1* and *hTERT* genes [41]. The most promising clone was then differentiated into hepatocyte-like cells using a three-step protocol [43]. The characterization of the immortalized hepatocyte-like cell line (imHC) was confirmed through the assessments of population doubling level (PDL), and hepatic markers [16]. HepG2, Huh7 and imHC were maintained in 1:1 DMEM/F12 media (Gibco) supplemented with 10% FBS (HyClone), 100 U/mL penicillin and 100 μg/mL streptomycin (HyClone), and 1% GlutaMAX (Gibco). All cell lines were maintained at 37°C, 5% CO_2_ in a humidified atmosphere. Cells were passaged every 3–5 days or whenever they reached 80% confluence, with 0.125% trypsin-EDTA (Gibco).

### Production of cell culture-derived HCV

Cell culture-derived HCV (HCVcc), genotype 2a, was prepared from pJFH-1 system [44]. The pJFH-1 plasmid was propagated in *E*. *Coli*, extracted using NucleoBond Xtra Midi plasmid (MACHEREY-NAGEL (MN)). After purification, the plasmids were linearized with a restriction enzyme *Xba*I, and used as a template for JFH-1 RNA synthesis. The full-length HCV RNA was synthesized with TranscriptAid T7 High Yield Transcription Kit (Thermo Fisher Scientific). HCVcc was produced through the transfection of JFH-1 RNA into either imHC or Huh7 cells using lipofectamine 3000 (Invitrogen). The supernatant was collected, and mixed with Lenti-X Concentrator (Clonetech, Takara Bio) at a ratio of 3:1 (clarified supernatant: Lenti-X Concentrator). The mixtures were incubated at 4°C overnight, centrifuged at 1500× *g* for 45 min at 4°C to discard supernatant. The pellet was gently re-suspended with FBS at dilution 1:100 of the original volume of supernatant. The 100× HCVcc was aliquoted and stored at -80°C. The HCV titer was about 1×10^7^ HCV genome equivalents /mL using the serial dilution of a known amount of pJFH-1 plasmid as a standard.

### Plasma-derived HCV inoculum

The study was conducted in accordance with the Declaration of Helsinki. Blood specimen of HCV-positive plasma was collected from chronic HCV-infected patients at Ramathibodi Hospital, Mahidol University during 1 Oct 2020–20 Nov 2022. The collection of leftover blood specimen was approved by the Ethics Committee on Research Involving Human Subjects of Ramathibodi Hospital (MURA2020/1545). All methods were carried out in accordance with relevant guidelines and regulations. All patient samples were analyzed anonymously, ensuring confidentiality and privacy rights throughout the study. The plasma-derived HCV genotypes 1 to 6 were determined by Auto-LiPA^™^ 48 machine (Fujirebio) with VERSANT^®^ HCV Genotype 2.0 Assay (LiPA) (SIEMENS). The viral load of plasma samples was measured by the Alinity m instrument (Abbott) with Alinity m HCV assay kit, with a limit of detection (LOD) at 12 IU/mL and a range up to 2.0 × 10^8^ IU/mL. The plasma samples containing viral load > 1.0 × 10^6^ IU/mL were collected for HCV inoculation, and the HCV-positive plasma (S1 Table) was subsequently aliquoted and stored at -80°C prior to inoculate.

### HCV infection to hepatocytes

Hepatocytes were seeded at 10^6^ cells /well (80% confluence) onto 6-well plate overnight. Cells were treated with 5 μM α-tocopherol (Sigma-Aldrich) and 1% lipid concentrate (Life Technologies) in 2 mL complete medium (Williams’ Media E (Sigma-Aldrich), 10% FBS, 100 U/mL penicillin, 100 μg/mL streptomycin, 1% GlutaMAX) for 24 h. The media was replaced by 1 mL of 4% PEG8000 and 0.25% Sodium Citrate in basal Williams’ Media E including antibiotic for HCV^+^-plasma; or 4% PEG8000 in basal Williams’ Media E including antibiotic for HCVcc. Then 50 μL of HCV^+^-plasma or the desired viral titer for HCVcc was added. At 24 h post-infection, the infected cells were washed thrice with 0.1% BSA in DPBS and reconstituted with complete media containing 1 μM α-tocopherol and 1% lipid concentrate. The media was renewed every 3 days post-infection (dpi) and harvested at 7 dpi, or maintained until 21 days.

### Quantitative PCR for host gene expression

The Human liver total RNA was purchased from Invitrogen™ (AM7960, Thermo Fisher Scientific). Total RNA was extracted from cell pellets by illustra RNAspin Mini RNA isolation kits (GE Healthcare, Asia Pacific). The RNA was converted to cDNA using the ImProm-II^™^ Reverse Transcription System (Promega) with the Oligo(dT)_15_ Primer. The cDNA was diluted to 1:10 before use. The primers used to quantify host mRNA included hepatocyte markers, HCV cell-associated receptors and essential host factors, host inflammatory markers, interferon-stimulated genes, apoptotic markers and metabolic activators of sofosbuvir (S2 Table). The qPCR was performed by KAPA SYBR^®^ FAST qPCR Kits (Kapa Biosystems) with 2 μL of 1:10 diluted cDNA and 0.2 μM primers, initiating at 95°C for 3 min, followed by 40 cycles of 95°C for 10 s, 60°C for 20 s, using CFX96 Touch Real-Time PCR Detection System (Bio-Rad). Each sample was measured in triplicate. PCR amplicons were confirmed by gel electrophoresis and melting curve analysis. Expression levels were calculated using either 2^-ΔCt^ method for relative expression or the 2^-ΔΔCt^ for fold-change normalized to the endogenous *GAPDH*. *miR-122* was calculated using *U6* for normalization.

### Antiviral treatments

To evaluate the response of infected imHC to anti-HCV agents (10 IU/mL IFN-α 2b (11343514, ImmunoTools), 20 μM ribavirin (R9644, Sigma-Aldrich), and 1 μM sofosbuvir (PSI-7977, Selleckchem). These anti-HCV agents were not cytotoxic at the selected concentrations as determined by MTT assay. The naïve imHC was infected with either HCVcc (genotype 2a) or HCV^+^-plasma from various genotypes. The treatment, either alone or in combination, started at 24 h post-infection and continued for 7 days. HCV positive-strand RNA level, as determined by qPCR, was used as an indicator of inhibitory response in comparison with the non-treatment condition.

### The detection of positive and negative strands of HCV RNA

To measure HCV viral load, the medium from HCV infected cells was concentrated by Lenti-X Concentrator (Clonetech, Takara Bio) and extracted for viral RNA using NucleoSpin^®^ RNA Virus extraction kit (MACHEREY-NAGEL, MN). To detect intracellular HCV RNA, total RNA was extracted from the pellet using illustra RNAspin Mini RNA isolation kits (GE Healthcare, Asia Pacific). Total RNA was converted to cDNA for HCV positive-strand template by the ImProm-II^™^ Reverse Transcription System (Promega) with random primers. HCV positive-strand RNA was quantitated by qPCR [45] using Luminaris qPCR Master Mixes (Thermo Fisher Scientific) with 2 μL undiluted cDNA and 0.4 μM primers. The primer pairs targeting HCV positive-strand RNA from either cultured media or cell pellets were utilized (S3 Table), resulting in product sizes of 150 bp and 270 bp, respectively. The confirmation of PCR amplicon for HCV positive-strand RNA involved melting curve analysis and gel electrophoresis. Differing from positive strand RNA, the qualitative detection of the negative-strand HCV RNA was achieved using the nested PCR technique [46] using KAPA SYBR® FAST qPCR Kits (Kapa Biosystems). Briefly, reverse transcription of the HCV negative-strand RNA was performed using the ImProm-II™ Reverse Transcription System (Promega) along with the specific primer (S3 Table), spanning the 5’UTR viral region [46]. The first PCR (PCR I) utilized 2 μL undiluted cDNA from previous step and 0.4 μM primer (S3 Table). Subsequently, 2 μL PCR I reaction mixture served as the template for the second PCR (PCR II), employing 0.2 μM specific primer pairs. The authenticity of PCR amplicons for HCV negative-strand RNA was confirmed through gel electrophoresis, revealing a product size of 251 bp. All qPCR reactions and nested PCR were performed using CFX96 Touch Real-Time PCR Detection System (Bio-Rad).

### Immunofluorescence staining

The imHC was seeded at 80% confluence and maintained in complete medium. Cells were fixed with 4% paraformaldehyde (PFA) for 30 min at room temperature, washed with PBS, incubated with blocking solution (3% BSA, 0.1% Triton X-100 in PBS) for 1 h at room temperature, and incubated with primary antibodies. Primary antibodies against hepatocyte markers were ALB (1:100 dilution, ab10241, Abcam), AFP (1:100 dilution, SC8399, Santa Cruz Biotechnology), Na^+^-taurocholate cotransporting polypeptide (NTCP;1:100 dilution, ab131084, Abcam), MRP2 (1:100 dilution, ab3373, Abcam) and HNF-4ɑ (1:100 dilution, SC6556, Santa Cruz Biotechnology). Primary antibodies against cell surface receptors were claudin-1 (1:100 dilution, ab15098, Abcam), occludin (1:100 dilution, ab31721, Abcam), CD81 (1:100 dilution, ab79559, Abcam), scavenger receptor type B class I (SR-BI) (1:100 dilution, NB400-104, Novus Biologicals), and EphA2 (1:100 dilution, 37–4400, Thermo Fisher Scientific). To detect HCV peptides at 7 days post-infection, infected cells were fixed and incubated with antibodies against viral non-structural and structural proteins. These included NS3 (1:100 dilution, ab65407, Abcam), NS5B (1:100 dilution, ab35586, Abcam), and HCV core antigen (1:100 dilution, ab2740, Abcam). The secondary antibodies goat anti-mouse conjugated with Alexa Fluor® 488 (1:500 dilution, A-11001, Invitrogen), goat anti-rabbit conjugated with Alexa Fluor^®^ 488 (1:500 dilution, A-11008, Invitrogen), goat anti-mouse conjugated with Alexa Fluor^®^ 568 (1:500 dilution, A-11004, Invitrogen), goat anti-rabbit conjugated with Alexa Fluor^®^ 568 (1:500 dilution, A-11011, Invitrogen), donkey anti-goat conjugated with Cy3 (1:500 dilution, AP180C, Merck). The secondary antibodies were applied against the corresponding unconjugated primary antibody. Cell nuclei were stained with Hoechst 33342 (Thermo Fisher Scientific). Mouse IgG2a, mouse IgG1, rabbit IgG and goat IgG were used as isotype controls. Fluorescent images of cells on 96-well black plate were detected and analyzed using an Operetta High-Content Imaging System (PerkinElmer) with a 40× objective lens from 12 randomly selected image fields (total number of analyzed cells > 2000), while non-structural and structural proteins of HCV in infected cells on 24-well plate were detected under the inverted fluorescent microscope (Olympus IX81) with a 20× objective lens.

### Flow cytometry

imHC and Huh7 were seeded onto a 6-well plate at 10^6^ cells /well and incubated overnight. Cells were pretreated with 5 μM α-tocopherol (Sigma-Aldrich) and 1% lipid concentrate (Life Technologies) in 2 mL complete medium (Williams’ Media E, Sigma-Aldrich), 10% FBS, 100 U/mL penicillin, 100 μg/mL streptomycin, 1% GlutaMAX) for 24 h. The hepatocytes were infected with HCVcc at a MOI of 1 or with HCV^+^-plasma. After 24 h, the infected hepatocytes were washed thrice with 0.1% BSA in DPBS and replaced with complete media containing 1 μM α-tocopherol and 1% lipid concentrate. Seven days post-infection, the infected cells were collected by trypsinization and washed thrice with 1 mL of PBS. Cell pellets were fixed with 4% paraformaldehyde for 30 min, permeabilized with 0.1% Triton-X solution for 20 min and washed with PBS. Subsequently, the cells were incubated with 3% BSA for 30 min to prevent non-specific binding, stained with anti-HCV-NS3 (1:200 dilution, ab65407, Abcam) at 4°C for 2 h, washed thrice with PBS, stained with goat anti-mouse conjugated with Alexa Fluor® 488 (1:500 dilution, A-11001, Invitrogen) at room temperature for 1 h, washed thrice with PBS, reconstituted with 1% FBS in PBS and analyzed using a BD FACSVerse flow cytometer.

### Western blot

Cells were collected from 6-well plate with 0.5 mL of 5 mM EDTA. Supernatants were removed and the 100 μL RIPA buffer (Merck) and fresh protease inhibitor cocktail (Thermo Fisher Scientific) were added. The samples were collected, and the protein concentration was measured using Pierce BCA Protein Assay Kit (Thermo Fisher Scientific). Electrophoresis was performed and proteins were transferred to polyvinylidene fluoride (PVDF) membranes (Merck). The membrane was blocked for non-specific binding and incubated with the primary antibodies against SEC14L2 (1:2,000 dilution, ab137013, Abcam) with 5% BSA in TBS/0.05% Tween 20 at 4°C overnight. Membranes were incubated for 1 h at room temperature with HRP-conjugated goat anti-rabbit antibody (1:5,000 dilution, ab97051, Abcam). For loading control, the blot was stripped, followed by probing with mouse anti-GAPDH antibody (1: 200,000 dilution, AM4300, Thermo Fisher Scientific) and HRP-conjugated goat anti-mouse secondary antibody (1: 5,000 dilution, ab97023, Abcam). The signal was developed by adding Luminata crescendo Western HRP substrate (Merck) and imaged with Omega Lum^™^ G Imaging System (Aplegen).

### Statistical analysis

All results of experiments were performed in triplicate and data were expressed as mean ± SD. Statistical analysis was performed with GraphPad Prism 10 (GraphPad Software, Inc., San Diego, CA). Normality test has been conducted prior to the difference test. Data were determined for differences using either Student’s unpaired t test or one-way ANOVA with either Dunnett or Tukey’s multiple comparisons test for parametric test. Mann Whitney test or Kruskal-Wallis test with Dunn’s multiple comparisons test were applied for non-parametric test. Statistical significance was considered when *p*-value < 0.05.

## Results

### The imHC exhibited hepatic phenotypes

**Fig 1:**
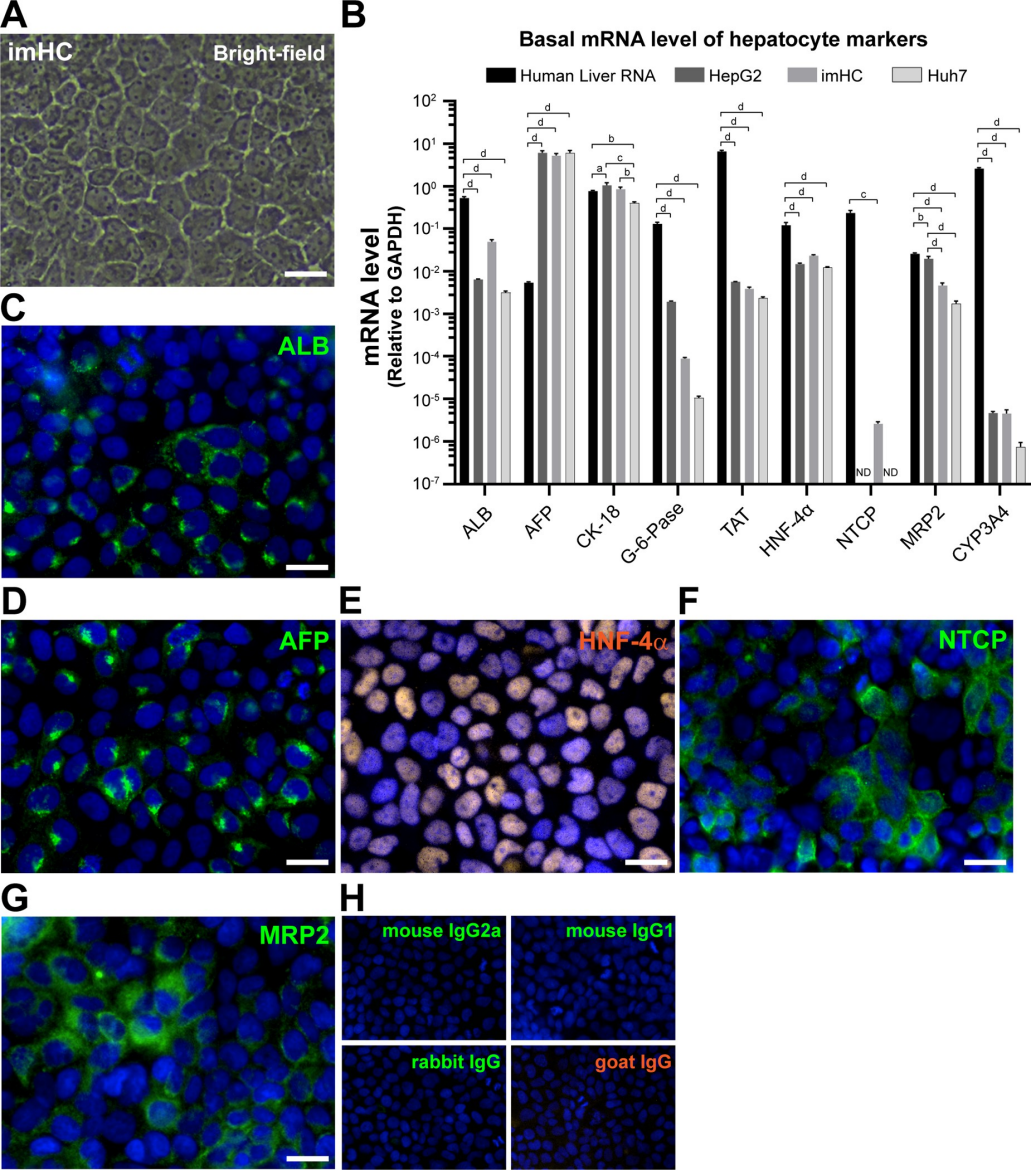
Hepatic phenotypes of immortalized hepatocyte-like cell (imHC). The attached imHC at confluence exhibited hepatocyte morphology: polygonal shape, granulated cytoplasm and large nucleus (A). The basal expression of hepatocyte markers in human total liver RNA, HepG2, imHC, and Huh7 were analyzed using real-time qPCR (B). Liver proteins, albumin (ALB) (C), α-fetoprotein (AFP) (D), hepatocyte nuclear factor-4-alpha (HNF-4ɑ) (E), Na^+^-taurocholate cotransporting polypeptide (NTCP) (F) and multidrug resistance-associated protein 2 (MRP2) (G) were evaluated using immunofluorescence. The nuclei were stained with Hoechst 33342. The isotype control antibodies were used as negative controls (H). Fluorescence images were taken by an Operetta High-Content Imaging System (PerkinElmer) with a ×40 objective lens. Scale bar = 50 μm. ND represented “not detected”, whereas a, b, c, and d represented statistical differences between cell lines with a *p*-value less than 0.05, 0.01, 0.001, and 0.0001 respectively.

imHC prepared from hMSCs, immortalized with human telomerase transcriptase (*hTERT*) and *Bmi-1* [42] was induced for hepatic differentiation [41]. imHC exhibited hepatocyte morphology: polygonal shape, granulated cytoplasm and large nucleus (Fig 1A). imHC was further characterized for hepatic phenotypes. These phenotypes (mRNA level (Fig 1B), protein level (Fig 1C–1G), and metabolic functions [16,31]) were compared with those of HepG2, Huh7 and human liver. We detected Na^+^-taurocholate cotransporting polypeptide (NTCP), a bile acid transporter into hepatocytes and multidrug resistance-associated protein 2 (MRP2), a bile canaliculi marker (Fig 1F and 1G) against the respective isotype controls (Fig 1H). These features positioned imHC as a more suitable option compared to other hepatoma cell lines, although it still fell short of the primary hepatocyte [16,31].

### imHC expressed all HCV-associated receptors and essential host factors

**Fig 2:**
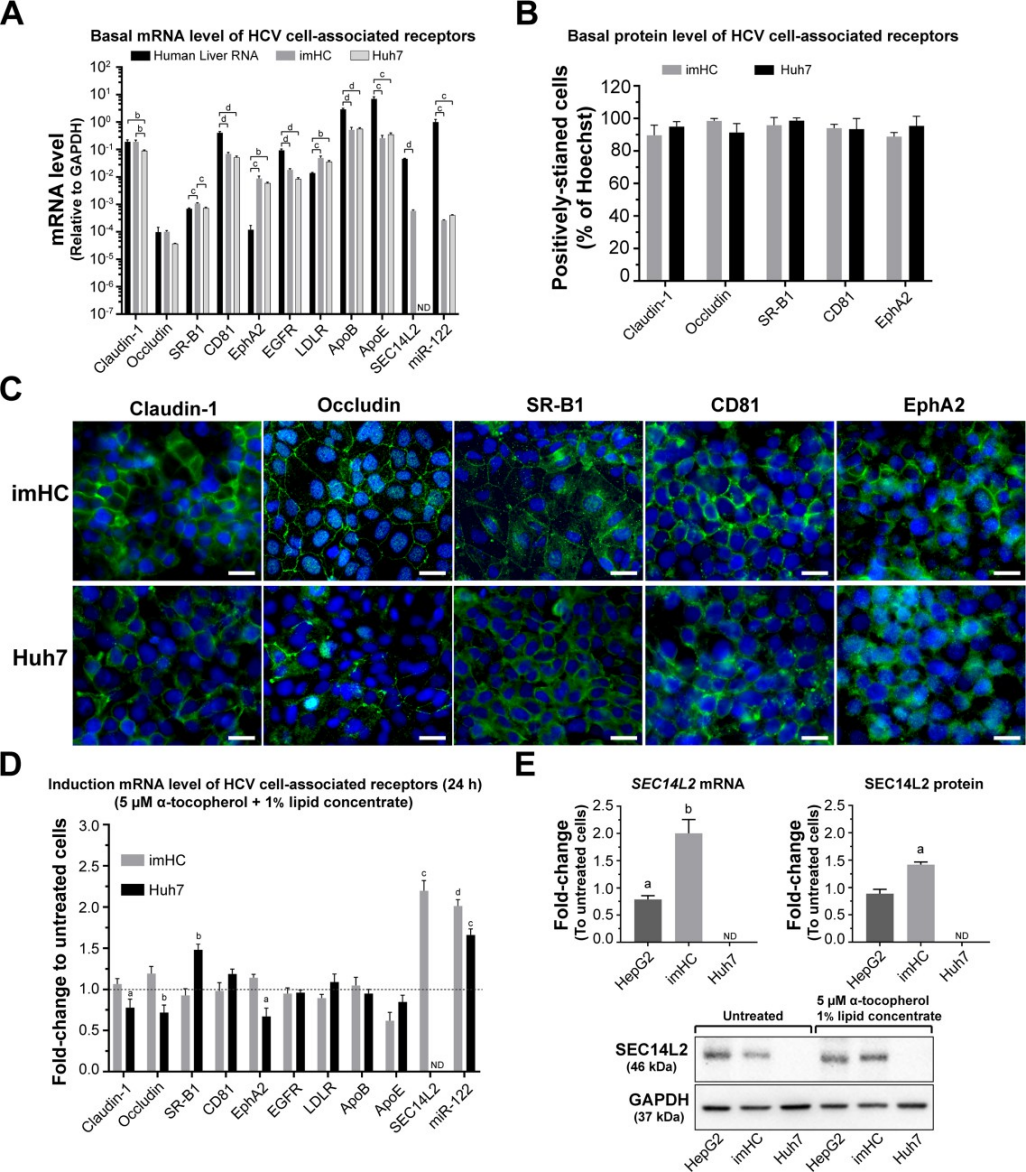
Identification of essential host factors and receptors for HCV in hepatocytes. Basal level of associated genes was determined using real-time PCR in human liver, imHC, and Huh7 (A). The quantitative analysis of Claudin-1, Occludin, SR-BI, CD81, and EphA2 in imHC and Huh7 were evaluated from 12 randomly selected image fields (total number of analyzed cells > 2000). The bar graph demonstrated the percentage of positively stained cells and the error bar represents SD (B). Cellular localizations of major HCV receptors (claudin-1, occludin, SR-BI, CD81 and EphA2) were detected using immunofluorescence staining (C). Hepatocyte nuclei were counterstained with Hoechst 33342. Fluorescence images were captured and analyzed by an Operetta High-Content Imaging System (PerkinElmer) with a ×40 objective lens with Scale bar = 50 μm. Both cell lines were treated with 5 μM α-tocopherol and 1% lipid concentrate for 24 h prior to the harvest for mRNA of HCV receptors and essential host factors (D). The SEC14L2 mRNA and protein were evaluated using qPCR and western blot analysis (E). ND represented “not detected”, whereas a, b, c, and d represented statistical differences between cell models or the treatments and their respective control with a *p*-value less than 0.05, 0.01, 0.001, and 0.0001 respectively.

To verify whether imHC could host HCV, the presence of cell-associated receptors for HCV entry and essential host factors for HCV replication were determined in imHC. imHC, Huh7, as well as human liver RNA were evaluated for the expression levels of cell surface receptors (*claudin-1*, *occludin*, *SR-BI*, *CD81*, *EphA2*, *EGFR* and *LDLR*), and host factors (*ApoB*, *ApoE*, *SEC14L2* and *miR-122*) (Fig 2A). *Claudin-1* expression in imHC was comparable to that in human liver RNA and significantly higher than that in Huh7 (*p* < 0.05), whereas the expression level of *occludin* was similar in all groups (Fig 2A). The expression levels of *CD81*, *EGFR*, *ApoB*, *ApoE*, and *miR-122* in imHC were identical to that in Huh7, but less than in human liver RNA (*p* < 0.05) (Fig 2A). In contrast, the RNA levels of *EphA2* and *LDLR* in both imHC and Huh7 were equivalent but were higher than those in human liver RNA (*p* < 0.05) (Fig 2A). Furthermore, the expression level of *SR-BI* in imHC was higher than that in Huh7 and human liver (*p* < 0.05) (Fig 2A). Importantly, *SEC14L2*, which was crucial for wild-type HCV replication [29], was exclusively expressed in imHC, but not in Huh7 cell (Fig 2A). More than 80% of Huh7 and imHC population carried HCV cell-associated receptors (Fig 2B). Localization of HCV-associated receptors for HCV entry (claudin-1, occludin, SR-BI, CD81 and EphA2) on imHC and Huh7 were visualized by immunofluorescence staining (Fig 2C). The proportion of positive cells were analyzed with Operetta High-Content Imaging System (PerkinElmer). The LDLR level in imHC and Huh7 was similar to previous reports [31,47]. To increase *SEC14L2* and *miR-122* expression [29,48], Huh7 and imHC were treated with 5 μM α-tocopherol and 1% lipid concentrate for 24 h (Fig 2D). The expression of these genes in imHC was increased by 2 folds. For Huh7, miR-122 was increased by 1.6 folds, but SEC14L2 was still undetectable after induction. HepG2 was used as a positive control for SEC14L2 detection [49]. After induction, HepG2 exhibited a slight decrease in both mRNA and protein levels of SEC14L2, whereas imHC exhibited 2-folds mRNA level and 1.4-folds protein level (Fig 2E). Taken together, imHC demonstrated equivalent cell-associated receptors for HCV entry to those in the classical host (Huh7). However, imHC exhibited superior essential host factors for accommodating wild-type HCV.

### imHC supported HCVcc production and infection

**Fig 3:**
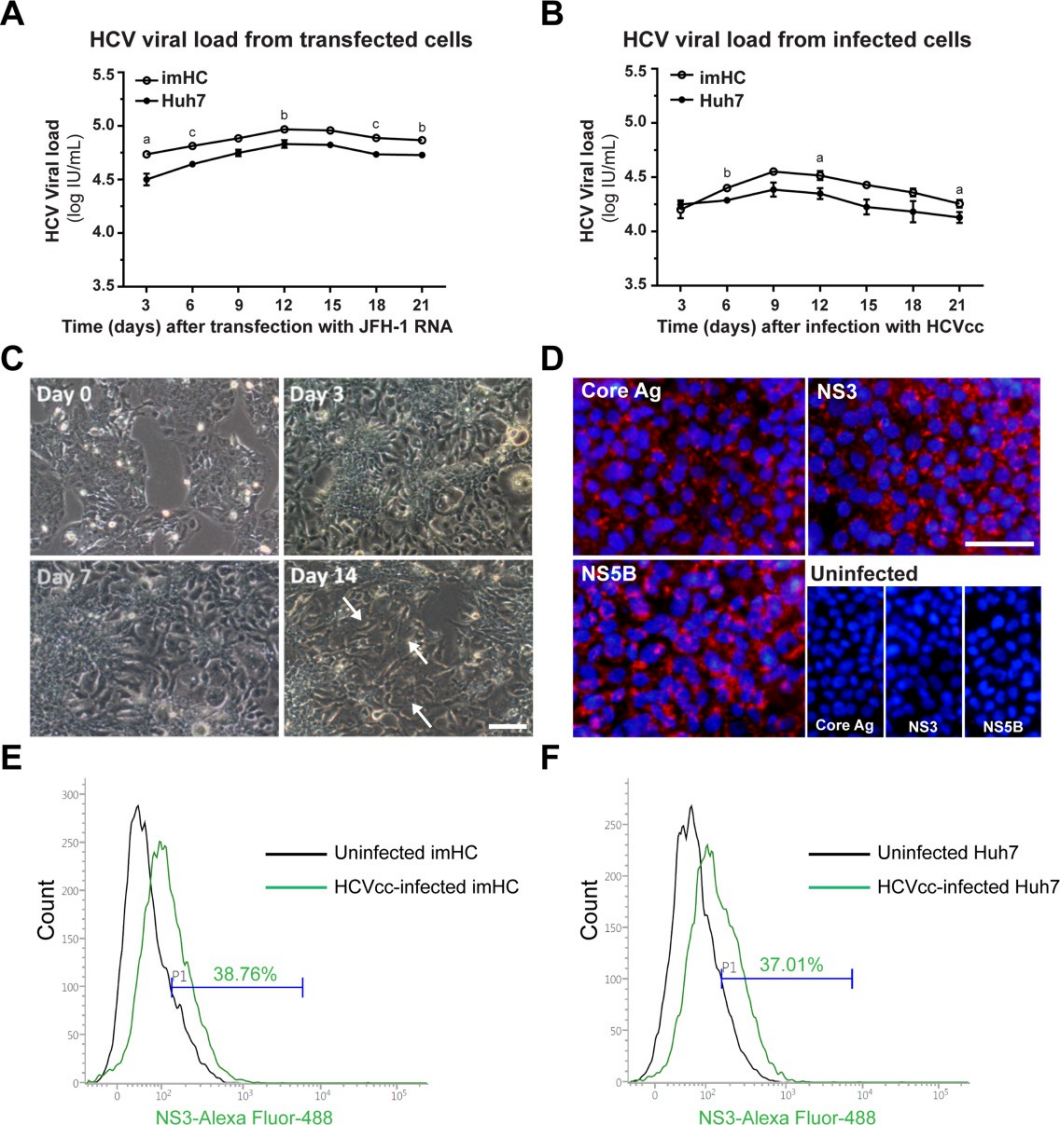
imHC as a host for HCVcc production and infection. The kinetics of HCV viral load after the transfection with JFH-1 RNA was demonstrated in imHC and Huh7 at 3-day intervals (A) or after the infection with HCVcc progeny at an MOI of 10 (B). Bright-field images were observed in imHC after HCVcc infection, and showed the CPE on day 14 (C). HCV proteins (Core Ag, NS3, and NS5B) in infected imHC were detected by IFA at 7 dpi (D). The infectivity of HCVcc in imHC (E) and Huh7 (F) was assessed via flow cytometry, targeting NS3-positive stained cells at 7 dpi. Scale bar = 50 μm, a, b, and c represented significant differences between cell lines at each time point with a *p*-value less than 0.05, 0.01, and 0.001, respectively.

Regarding HCVcc production, imHC and Huh7 were transfected with JFH-1 RNA. Conditioned medium from imHC and Huh7 was harvested every 3 days for HCV viral load detection. The viral load from imHC was higher than that of Huh7 in all sampling times. The maximal viral load from imHC was over 10^5^ IU/mL, while that of Huh7 was merely 6.8 × 10^4^ IU/mL (*p* < 0.01) at 12 dpi (Fig 3A). For HCVcc infection, the conditioned medium from infected cells was taken from the progeny infection every 3 days for viral load. The viral load in imHC was again higher in Huh7 all sampling times. The highest viral load peaked at day 9 post-infection (Fig 3B) with the viral load from imHC reached 3.57 × 10^4^ IU/mL, surpassing Huh7’s viral load of 2.45 × 10^4^ IU/mL. HepG2 served as the control for both transfection and infection experiments. However, it did not support HCV infection in any circumstance (data not shown). Regarding the infectivity of HCVcc progeny into naive imHC, cytopathic effect (CPE) was observed after two weeks (Fig 3C), while the HCV proteins (Core Ag, NS3, and NS5B) were detected by IFA at 7 dpi (Fig 3D). The infectivity of HCVcc in imHC and Huh7, subsequent to infection with HCVcc at an MOI of 1, was evaluated using flow cytometry. Results revealed 38.76% and 37.01% of NS3-positive stained cells at 7 dpi for imHC and Huh7 cells respectively (Fig 3E and 3F). Therefore, imHC served as an efficient host for the propagation and infection of HCVcc (JFH-1 strain).

### imHC allowed full life cycle of HCV from clinical isolates

**Fig 4:**
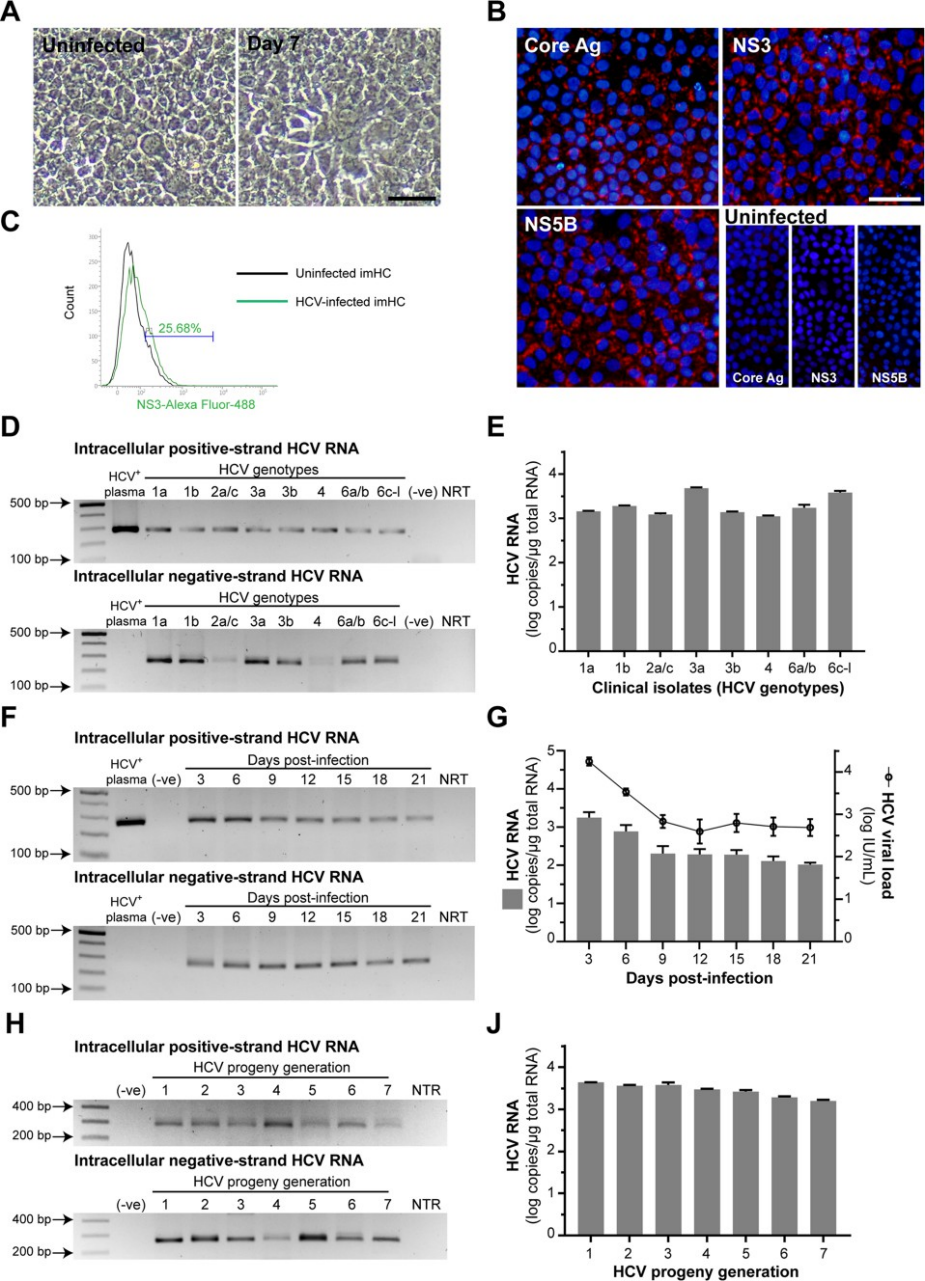
imHC allows full life cycle of plasma-derived HCV *in vitro*. imHC was infected with several HCV genotypes derived from plasma. Bright-field images of imHC morphology were observed at 7 days post-infection (dpi) with sample RAVL09 (A). HCV proteins (core Ag, NS3 and NS5B) in infected imHC with sample RAVL09 were detected using IFA on day 7 dpi (B). Scale bar = 50 μm. The infectivity of HCV in imHC, following infection with plasma sample RAVL09, was evaluated using flow cytometry, with emphasis on NS3-positive stained cells at 7 dpi (C). Several HCV genotypes obtained from plasma samples (sample IDs from RAVL01 to RAVL08) were inoculated into imHC. After 7 dpi, HCV RNA was harvested from cell pellets for positive and negative RNA strands detection. The PCR products were separated and visualized by gel electrophoresis. (D). Intracellular HCV RNA of pan-genotype HCV in imHC was quantified by qPCR (E). The kinetics of HCV RNA levels in imHC after infection with sample RAVL09 were demonstrated every 3 days (F), and the intracellular HCV RNA and HCV viral load were measured up to 21 dpi (G). HCV progeny collected from conditioned medium after infection with sample RAVL10 can infect the naïve imHC. Positive and negative RNA strands were detected in cell pellet from each generation of HCV (H). Intracellular HCV RNA level in each HCV generation was quantified (J).

To examine HCV infectivity from clinical isolates, HepG2 and Huh7 were used as negative controls. HepG2 was not susceptible to HCV infection due to the absence of CD81 [38], while Huh7 lacks an essential host factor necessary for replication of clinically-isolated HCV [29]. HepG2, Huh7 and imHC were infected with various HCV genotypes from HCV^+^-plasma. After infection, total RNA was extracted from cell pellets to detect HCV-positive and negative RNA strands. HCV-positive RNA strand indicated HCV entry and production, while the negative RNA strand represented HCV replication. Both positive and negative RNA strands were undetectable in HepG2 (S1A and S1B Fig). In Huh7, the positive RNA strand of some HCV genotypes was detected (S1C Fig), but the negative strand RNA was undetectable (S1D Fig). This demonstrated that Huh7 allowed plasma-derived HCV entry but not replication. The morphology of plasma-derived HCV-infected imHC (Fig 4A) as well as the presence of structural and nonstructural HCV proteins (Core Ag, NS3, and NS5B) were observed at 7 days post-infection (Fig 4B). Furthermore, the infectivity of the clinical HCV isolate in imHC resulted in 25.68% of NS3-positive stained cells at 7 dpi, as determined by flow cytometry (Fig 4C). In imHC, both HCV-positive and negative RNA strands were found after the infection with all studied HCV genotypes (Fig 4D). Besides, the intracellular level of HCV-positive strand RNA in imHC exceeded 3 log copies/μg total RNA in all genotypes (Fig 4E). The kinetics of HCV replication using intracellular HCV RNA and HCV viral load (Fig 4F and 4G) suggested that imHC can support a complete HCV replication and persistence of infection from clinical isolates. The viral progeny in conditioned medium from infected cells could infect naïve imHC. Both positive and negative-stranded HCV RNA were detected in cell pellet from several generations of HCV progeny (Fig 4H–4J). These indicated that imHC could serve as a competent hepatocyte model for clinical HCV infection.

### The alteration of host cells in response to HCV infection

**Fig 5:**
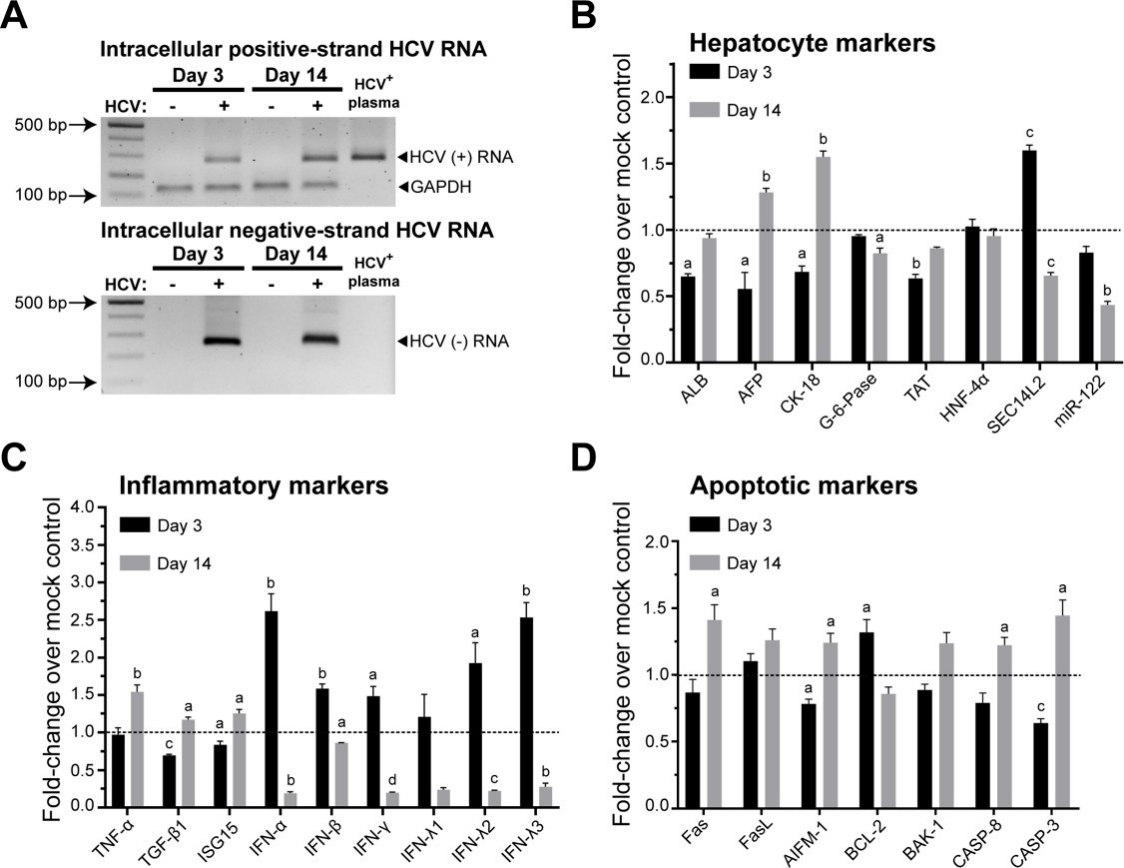
Investigation of host cell response to patient-derived HCV infection. imHC was infected with HCV^+^-plasma (sample RAVL11, genotype 1b) and maintained for 14 dpi. The intracellular positive and negative HCV RNA strands were detected in infection or mocked infection in imHC on 3 and 14 dpi (A). The expressions of hepatocyte markers (B) inflammatory markers (C) and apoptotic markers (D) were determined by qPCR in infected imHC or mock-infected imHC on 3 and 14 dpi. The a, b, c, and d represented significant difference between the infection and the mock control in each time point with a *p*-value less than 0.05, 0.01, 0.001, and 0.0001 respectively.

imHC and Huh7 were infected with HCV from either clinical isolate or HCVcc for 14 dpi. The intracellular HCV positive and negative-stranded RNA were determined in mock infection and HCV-infected imHC on 3 and 14 dpi. The intracellular HCV positive-stranded RNA was initially detected on 3 dpi and increased until 14 dpi (Figs 5A and S2A). During the infection, the integrity of the expressions of hepatic genes expression, inflammatory and apoptotic markers in host cells was observed. The alteration of hepatic genes in response to HCV infection included the reduction of *ALB*, *AFP*, *CK-18*, and *TAT* expression on 3 dpi. *ALB* decreased to 0.64 folds on 3 dpi and recovered on 14 dpi. *AFP* and *CK-18* decreased to 0.55 and 0.68 folds respectively at early stage and reversed to 1.28 and 1.55 folds on 14 dpi. *TAT* decreased to 0.63 folds after infection (Fig 5B). HCVcc infection toward imHC decreased *G-6-Pase* and *HNF-4α* expressions to 0.15–0.5 folds respectively on 3 dpi with full recovery of *HNF-4α* expression on 14 dpi (S2B Fig). On the other hand, *miR-122* decreased after the infection with either HCVcc or clinical isolate (Figs 5B and S2B). The expression of *SEC14L2* increased to 1.60 folds on 3 dpi and reversed to 0.65 folds on 14 dpi after clinical isolate infection (Fig 5B). For Huh7 cell, the intracellular HCV positive-strand RNA was increased on 3 dpi after HCVcc infection and declined on 14 dpi (S2E Fig). *ALB*, *G-6-Pase*, and *TAT* decreased over early dpi but were reversed to 1.4 folds on 14 dpi (S2F Fig). The *AFP* expression in Huh7 was not affected by HCV infection, while *CK-18* and *miR-122* increased to 1.4 folds. *HNF-4α* slightly decreased in early dpi and recovered on 14 dpi (S2F Fig).

The expression of inflammatory cytokines was evaluated using imHC (Figs 5C and S2C) and Huh7 (S2G Fig) after HCV infection. *TNF-α* and *TGF-β1* were increased to 1.25–1.5 folds on 14 dpi in both cells. *ISG15* was also increased to 1.25 folds on 14 dpi in imHC, but in Huh7, it showed an augmentation on 3 dpi and declined on 14 dpi. *IFN-α*, *IFN-β*, *IFN-γ*, *IFN-λ1*, *IFN-λ2* and *IFN-λ3* increased to 1.25–2.5 folds on 3 dpi and declined on 14 dpi in both cells (Figs 5C, S2C and S2G). For apoptosis genes, imHC responded to HCV infection by increasing apoptotic markers (Figs 5D and S2D), whereas Huh7 resisted apoptosis by enhancing *BCL-2* gene expression (S2H Fig). The expression of Fas/FasL, a ligand-induced apoptosis (extrinsic pathway), was increased in imHC after infection with either HCVcc or clinical isolates (Figs 5D and S2D). For Huh7 cell, *Fas* increased on 14 dpi and *FasL* rose on both times after HCVcc infection (S2H Fig). In imHC, the intrinsic apoptosis pathway expression profiles were similar in both conditions of infection (Figs 5D and S2D). For intrinsic apoptotic pathway, the anti-apoptotic gene, *BCL-2* decreased while pro-apoptotic markers *AIFM-1* and *BAK-1* increased after two weeks of HCV infection. The *Caspase-8* and *Fas* expressions increased to 1.25–1.3 folds, while *Caspase-3* expression, an intrinsic apoptosis pathway marker, significantly increased to 1.5 folds (Figs 5D and S2D) that would induce apoptosis. However, the expression of intrinsic apoptosis genes in Huh7 displayed incoherent fashion (S2H Fig). HCVcc infection to Huh7 could not induced intrinsic apoptosis pathway (S2H Fig), although pro-apoptotic markers (*AIFM-1* and *BAK-1*) increased after 14 dpi. The *Caspase-8* and *Caspase-3* expressions were slightly increased on 3 dpi but declined on 14 dpi that suggested the failure to induce apoptosis. In contrast, the anti-apoptotic factor, *BCL-2* was elevated starting from 3 dpi and peaked on 14 dpi in Huh7 (S2H Fig).

### The response of infected-imHC to anti-HCV agents

**Fig 6:**
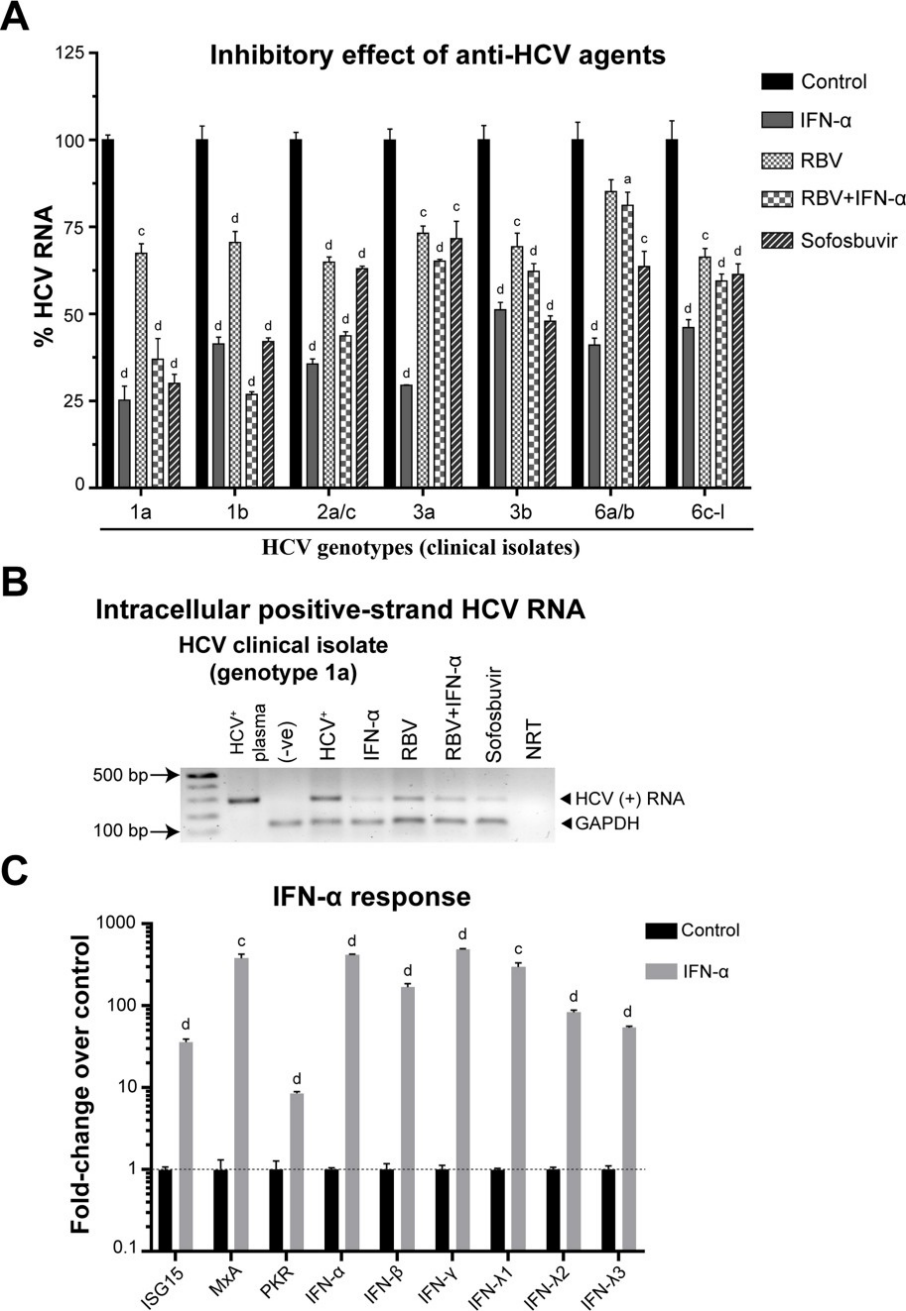
imHC served as a competent HCV model for new antiviral drug development. imHC was infected with HCV from clinical isolates of various genotypes (sample IDs of RAVL03, RAVL07, RAVL08, and RAVL13 to 15) and treated with anti-HCV agents: IFN-α (10 IU/mL), ribavirin (20 μM) and sofosbuvir (PSI-7977, 1 μM) or drug combinations for 7 days. The HCV positive RNA strand level in infected cells served as an indicator for drug response (A). The representative PCR products of HCV positive RNA strand and GAPDH were shown (B). The induction of antiviral signaling genes in response to IFN-α was investigated in imHC infected with HCV sample RAVL13 (genotype 1b) (C). The up-regulation of interferon-stimulating genes (ISGs) and IFN type I-III after IFN-α treatment was plotted against those without IFN-α treatment. The a, b, c, and d represented significant difference between the treatments and their respective control with a *p*-value less than 0.05, 0.01, 0.001, and 0.0001 respectively.

All anti-HCV agents at the given doses were examined for their cytotoxicity. In imHC, the 50% cytotoxic concentration (CC_50_) of IFN-α, ribavirin (RBV) and sofosbuvir were > 1000 IU/mL, 87.23 μM and 66.08 μM respectively (S3 Fig). The anti-HCV activity was quantified through the level of positive HCV RNA strand from infected hepatocytes using qPCR (Figs 6A, 6B and S4C). For HCVcc infection, treatment with various concentrations of IFN-α in HCVcc-infected imHC demonstrated a dose-dependent response, leading to a reduction in HCV RNA levels by up to 85%, surpassing the reduction observed in HCVcc-infected Huh7 cells (S4A Fig). In contrast, treatment with varying concentrations of RBV in HCVcc-infected Huh7 cells exhibited a dose-dependent response, resulting in a reduction of HCV RNA levels greater than that observed in HCVcc-infected imHC (S4B Fig). In the same way, the single dose treatment of HCVcc-infected Huh7 and HCVcc-infected imHC with IFN-α, sofosbuvir or the combination (IFN-α+RBV) decreased HCV RNA about 50%, whereas RBV displayed poor anti-HCV activity in both imHC and Huh7 (S4D Fig). In summary, HCVcc-infected imHC displayed a superior response to IFN-α, whereas HCVcc-infected Huh7 cells exhibited a more favorable response to RBV. For plasma-derived HCV, IFN-α treated infected-imHC decreased HCV RNA by 50–75% of all genotypes. The combination of IFN-α+RBV displayed higher anti-HCV activity against genotype 1b than did the single IFN-α treatment. The RBV treatment slightly decreased intracellular HCV RNA by 30–35% in all genotypes indicating the patient-derived HCV exhibited a partial response to RBV treatment. Sofosbuvir decreased intracellular HCV RNA of genotype 1a and 1b by 70–75%. However, sofosbuvir moderately decreased HCV RNA of genotype 2a/c, 3a, 6a/b and 6c-l by 30–40%, indicating poor susceptibility of these strains to sofosbuvir (Fig 6A and 6B). IFN-α treatment exhibited superior anti-HCV activity in both HCVcc and clinical isolates. This might be attributed to interferon-stimulated genes (ISGs) that exerted numerous antiviral functions.

The interferon-stimulated gene (ISGs) expression in infected imHC and Huh7 after IFN-α treatment was investigated. imHC was infected with HCV (RAVL13, genotype 1b) for 7 days and treated with or without IFN-α (Fig 6C), while IFN-α response in HCVcc infection was conducted in both imHC and Huh7 cell lines (S4E Fig). The IFN type I-III were significantly increased in both Huh7 and imHC after IFN-α treatment, but imHC expressed higher *ISG15* and *MxA* than did Huh7 (Figs 6C and S4E). For drug biotransformation, sofosbuvir is a prodrug requiring intracellular metabolic activators [50], *CES1*, *CatA*, *HINT1*, *UMP-CMPK*, and *NDPK* to become an active triphosphate form. The basal mRNA expression of metabolic activators revealed that *CES1*, the first key enzyme in sofosbuvir metabolism was higher expressed in imHC than in Huh7 (S5A Fig). Conversely, the two final enzymes in this pathway, UMP-CMPK and NDPK, exhibited higher expression in Huh7 than in imHC (S5A Fig). After incubating imHC with 0, 1, and 5 μM sofosbuvir, the expression of *CES1* and *CatA* increased with a dose-dependent manner. *HINT1*, *UMP-CMPK*, and *NDPK* were decreased in imHC at 0 and 1 μM and touched the basal level after 5 μM sofosbuvir treatment (S5B Fig). In contrast, the induction of metabolic genes was not observed in Huh7 and their expressions were diminished by sofosbuvir (S5C Fig). To assess the efficacy of sofosbuvir, imHC and Huh7 cells were infected with HCVcc and subsequently treated with varying concentrations of sofosbuvir for 7 days. The antiviral efficacy of sofosbuvir treatment was notably stronger in imHC compared to Huh7 cells (S5D Fig). The results bestowed the potential of using imHC for drug biotransformation study of several antiviral agents, especially nucleos(t)ide analogs (NAs).

## Discussion

The development of new anti-HCV agents relies on the availability of suitable screening models that properly mimic natural HCV infection. HCVcc developed from JFH-1 strain (genotype 2a) allowed entire HCV life cycle in Huh7 cells without cell-culture adaptive mutation [10]. Early HCV research, therefore, relied on HCVcc (JFH-1 strain) in Huh7 cell or its derivatives (Huh7.5 and Huh7.5.1) [12–14]. Other HCV genotypes could not propagate in Huh7 cells due to cell tropism and the restrict host range. The direct-acting antiviral agents (DAAs) were confronted with HCV resistance strains due to natural selection, treatment failure and clinical conditions [11]. A hepatocyte model capable of naturally accommodating resistant strains found in infected patients would accelerate the development of novel antiviral agents. *SEC14L2* (SEC14-like protein 2), or tocopherol-associated protein 1 (TAP1), could enable replication of pan-genotype HCV in Huh7.5 cells [29]. SEC14L2-expressing Huh-7.5 cells allowed replication of HCV derived from all clinical isolates [30]. Our model, imHC, was established from human bone marrow mesenchymal stem cells (hMSCs), exhibited robust expression of hepatocyte markers with putative functions [41]. We have verified that imHC could serve as an alternative hepatocyte model for HCV.

HCV engaged in a multistep process upon entering hepatocytes: beginning with its binding to apolipoproteins, HSPG, and LDLR [36]. Subsequently, it interacted with SR-BI, CD81, as well as tight junction proteins like Claudin-1 and Occludin. These interactions paved the way to internalization of HCV via clathrin-dependent endocytosis [35]. Moreover, EGFR and EphA2 played notable roles in subsequent stages of HCV entry [34]. Notably, ApoB and ApoE were implicated in the initial production of HCV particles in Huh7 [40], as well as in the subsequent assembly and maturation of HCV particles [37]. Through our analysis, we observed high mRNA and protein expression of HCV cell-associated receptors, which were crucial for HCV entry. Similarly, host factors (*ApoE* and *ApoB*) responsible for HCV particle production and maturation, displayed comparable expression levels in imHC and Huh7. *miR-122*, an essential host factor, was pivotal in HCV RNA replication [38,39], by binding to two sites in the 5′-UTR of the HCV genome leading to subsequent replication and translation [51]. Treatment with α-tocopherol and lipid concentrate resulted in significantly higher levels of *miR*-122 in imHC than in Huh7, while preserving *SEC14L2* expression. Notably, imHC expressed both *SEC14L2* mRNA and protein, facilitating pan-genotype HCV infection from clinical isolates [29,30]. We proposed that imHC surpasses classical Huh7 as a host for clinical HCV propagation with no requirement for ectopic host factors.

Huh7.5 and Huh7.5.1 were developed as alternatives to PHHs, exhibiting the capacity to produce HCVcc titers up to 10^5^ IU/mL [14]. However, these cells carried a mutation in RIG-I that was a key sensor in viral genome recognition for host immune response [12]. imHC natively allowed higher HCVcc production (10^5^ IU/mL) than did Huh7 after JFH-1 RNA transfection. The progeny virions could infect naïve imHC with a viral load of 3.57 × 10^4^ IU/mL. The JFH-1 and Huh7 cell systems allowed only HCV genotype 2a for drug screening. Clinically isolated HCV has been classified into six major genotypes and various subtypes [52] that required proper host models. These clinically isolated HCV failed to propagate in Huh7 cells due to their deficiency in *SEC14L2* [48]. In contrast, PHHs and iHLCs demonstrated the ability to produce approximately 10^4^ copies/μg total RNA after infection with clinical isolates [48,53]. In our study, imHC demonstrated support for HCV entry and replication across all genotypes, including 1a, 1b, 2a/c, 3a, 3b, 4, 6a/b, and 6c-l. The levels of HCV-positive RNA strand in infected imHC consistently exceeded 10^3^ copies/μg total RNA for all genotypes. Upon entering the imHC cells, HCV virions were protected from lipid peroxidation through increasing SEC14L2 expression via vitamin E treatment during infection [29]. Subsequently, *miR*-122 played a key role in facilitating HCV RNA replication and translation [38,39]. The process also involved ApoB and ApoE in the production of HCV particles [40]. Lastly, the assembly and secretion of infectious HCV particles closely mimicked the VLDL secretion pathway in hepatocytes [37].

The alterations of hepatic gene expression in infected-imHC and infected Huh7 were detected. *AFP* and *CK-18* were upregulated after of infection for 2 weeks. This observation was consistent with the elevated serum levels of CK-18 and AFP often observed in chronic viral hepatitis C (cHCV) and hepatocellular carcinoma (HCC) [54]. The reduction in albumin (ALB) levels following viral infections was documented in dengue virus infection, with a notable decrease in ALB expression observed on day 3 post-infection [55]. Host cell innate immune response played an important role in antiviral activity [56]. HCV infection triggered IFN type I-III expression at early infection via RIG-I mediated activation of IFN [56]. After 2 weeks of infection, the expression of IFN type I-III was significantly suppressed in imHC, likely due to the interaction of HCV proteins with host cellular signaling proteins, thereby influencing the innate immune response [56]. *ISG15* was reported as a proviral host factor for HCV [57], and its expression level positively correlated with the HCV replication level in agreement with our results. However, *TNF-α* and *TGF-β* were increased instead of IFN and correlated with apoptosis when the cellular innate response was unable to eliminate intracellular viral pathogen [58]. Apoptosis was triggered in imHC after 2 weeks of HCV infection. Nonetheless, the expression of inflammatory markers in HCVcc-infected Huh7 exceeded that in imHC. Once Huh7 effectively curtailed HCV propagation through the activation of the cellular innate response, it exhibited resistance to apoptosis by upregulating BCL-2. Clinical data supported this observation, showing significant BCL-2 accumulation in the livers of individuals with chronic HCV infection, with its levels subsequently reduced by IFN-2b plus ribavirin therapy [59].

The sensitivity of anti-HCV agents (IFN-α, ribavirin, and sofosbuvir) in imHC against both HCVcc and clinical isolates was evaluated in our study. Huh7 exhibited a notable response to classical anti-HCV agents, particularly ribavirin treatment against HCVcc, whereas imHC demonstrated a robust response to IFN-a treatment, eliciting activation of interferon-stimulated gene (ISGs) expression. imHC exhibited a better response to sofosbuvir through the conversion to its active triphosphate form by intracellular metabolic activators (CES1, CatA, HINT1, UMP-CMPK, and NDPK) [50]. The anti-HCV activity was investigated in infected cells with clinical HCV genotype 1a, 1b, 2a/c, 3a, 3b, 6a/b and 6c-l. The IFN-α treatment exhibited higher antiviral activity than did DAA for all genotypes. On the contrary, the combination of IFN-α and RBV was less effective than was IFN-α alone. Prolonged RBV treatment can lead to RNA mutations, resulting in variations of HCV proteins from the wild-type, potentially enabling HCV to evade the immune response and develop resistance to antiviral agents [60]. Treatment of imHC with IFN-α induced the expression of interferon-stimulated genes (ISGs) that mimicked natural cellular innate response. Additionally, our findings revealed that ISGs induced by IFN-α after HCV infection [53], were higher in imHC being infected with clinical isolate than with HCVcc. In summary, this study demonstrated that imHC could serve as a competent host for all known HCV genotypes. imHC could host full life cycle of HCVcc (JFH-1) and patient-derived HCV. This model could surpass Huh7 and JFH-1 system, which was restricted to HCV genotype 2a exclusively. With the increasing prevalence of anti-HCV drug resistance in patients, the use of imHC could serve as an excellent platform for evaluating anti-HCV agents against clinical samples.

## Conclusion

Here, we have established a hepatocyte model for clinical HCV infection. Our model can support the full-life cycle of HCV from both laboratory strain HCVcc (JFH-1) and clinical isolates with genotypes 1a, 1b, 2a/c, 3a, 3b, 4, 6a/b, and 6c-l. HCV infection in imHC caused alterations in host hepatic markers, which were subsequently counteracted by the host’s cellular innate response that led to apoptosis. This HCV-cell culture model would fulfill the study of HCV pathobiology, host cellular innate response and antiviral screening.

## Supporting information

S1 Fig The infectivity of pan-genotype HCV derived from clinical isolates. HepG2 and Huh7 were used as negative controls. HepG2 and Huh7 were infected with various HCV^+^-plasma genotypes (RAVL01 to RAVL08). After 24 h post-infection, hepatocytes were washed thrice with 0.1% BSA in DPBS and cultured for 7 d. The RNA was extracted from infected cell to detect HCV-positive and -negative RNA strands by RT-PCR. PCR products were visualized by gel electrophoresis. HCV-positive and -negative RNA strands were not detected in HepG2 (A, B). HCV-positive RNA strand was found in some HCV genotypes, (C), but no HCV-negative RNA strand (D), was detected in Huh7. The extracted RNA from HCV-positive plasma and HCV genotype 1b-infected imHC were used as positive controls for HCV-positive RNA strand and -negative RNA strand, respectively.
(TIF)

S2 Fig The responses of host cells to HCVcc infection. Hepatocytes were infected with HCVcc for 14 days. The intracellular HCV positive and negative RNAs were evaluated in imHC (A) and Huh7 (E) on 3 and 14 dpi. The expressions of hepatocyte markers in imHC (B) and Huh7 (F), inflammatory markers in imHC (C) and Huh7 (G), apoptotic markers in imHC (D) and Huh7 (H) were quantitated with qPCR after HCV or mock infection on 3 and 14 dpi. a, b, c, and d represented significant difference between the infection and the control with a *p*-value less than 0.05, 0.01, 0.001, and 0.0001 respectively.
(TIF)

S3 Fig The cytotoxicity of anti-HCV drugs in hepatocyte using MTT assay. imHC (3 × 10^4^ cells per well) were incubated with IFN-α (A), ribavirin (B), or sofosbuvir (C) for 7 days. The % viability of host cells were plotted as mean ± SD from eight replicates of each concentration. The 50^th^ percentile cytotoxic concentrations (CC_50_) in imHC were > 1000 IU/mL for IFN-α, 87.23 μM for ribavirin, and 66.08 μM for sofosbuvir.
(TIF)

S4 Fig The inhibition of HCV RNA by anti-HCV agents in HCVcc-infected hepatocytes. imHC and Huh7 were infected with HCVcc (genotype 2a) at MOI 1 and subsequently treated with anti-HCV agents for 7 days. The intracellular HCV positive RNA level in infected cells was determined by qPCR. The anti-HCV activity of IFN-α (A) and ribavirin (B) was evaluated. The PCR products of intracellular HCV positive RNA and GAPDH were displayed (C). The reduction of HCV RNA by IFN-α (10 IU/mL), ribavirin (20 μM), sofosbuvir (PSI-7977, 1 μM), or their combinations was investigated (D). The induction of antiviral genes response to IFN-α treatment was investigated in HCVcc-infected cells (E). These genes were plotted as fold-change over the corresponding untreated group. Abbreviations: interferon-stimulated gene 15 (*ISG15*), human myxovirus resistance protein 1 (*MxA*), protein kinase R (*PKR*), interferon-alpha (*IFN-α*), interferon-beta (*IFN-β*), interferon-gamma (*IFN-γ*), and interferon-lamda (*IFN-λ*). a, b, c, and d represented significant difference between cell lines or the treatments and their respective control with a *p*-value less than 0.05, 0.01, 0.001, and 0.0001 respectively.
(TIF)

S5 Fig Induction of intracellular metabolic activators by sofosbuvir. imHC and Huh7 were treated with sofosbuvir for 7 days. The expression of *CES1*, *CatA*, *HINT1*, *UMP-CMPK*, and *NDPK* in hepatocytes was evaluated as basal levels (A). These genes were increased in imHC (B) and were decreased in Huh7 (C) after treated with 0, 1, and 5 μM sofosbuvir. The gene expression was shown as fold-changes over the corresponding untreated groups. imHC and Huh7 were infected with HCVcc (genotype 2a) at MOI 1 and subsequently exposed to various concentrations of sofosbuvir for 7 days. The intracellular positive-stranded HCV RNA level in infected cells was evaluated as drug response compared to untreated control (D). The 50^th^ percentile of inhibition concentrations (IC_50_) of sofosbuvir was 1.06 μM and 2.41 μM for imHC and Huh7 respectively. a, b, c, and d represented significant difference between cell lines or the treatments and their respective control with a *p*-value less than 0.05, 0.01, 0.001, and 0.0001 respectively.
(TIF)

S1 Table Data of HCV^+^-plasma samples. (DOCX)

S2 Table Primer sets and conditions used in qPCR for host gene expression. (DOCX)

S3 Table Primer sets and conditions used to detect HCV RNA. (DOCX)
