# Supplementary figures and images for: Immortalized hepatocyte-like cells: A competent hepatocyte model for studying clinical HCV isolate infection
Source: PLoS One. 2024 May 13;19(5):e0303265. doi: 10.1371/journal.pone.0303265 (PMC11090328; doi:10.1371/journal.pone.0303265)

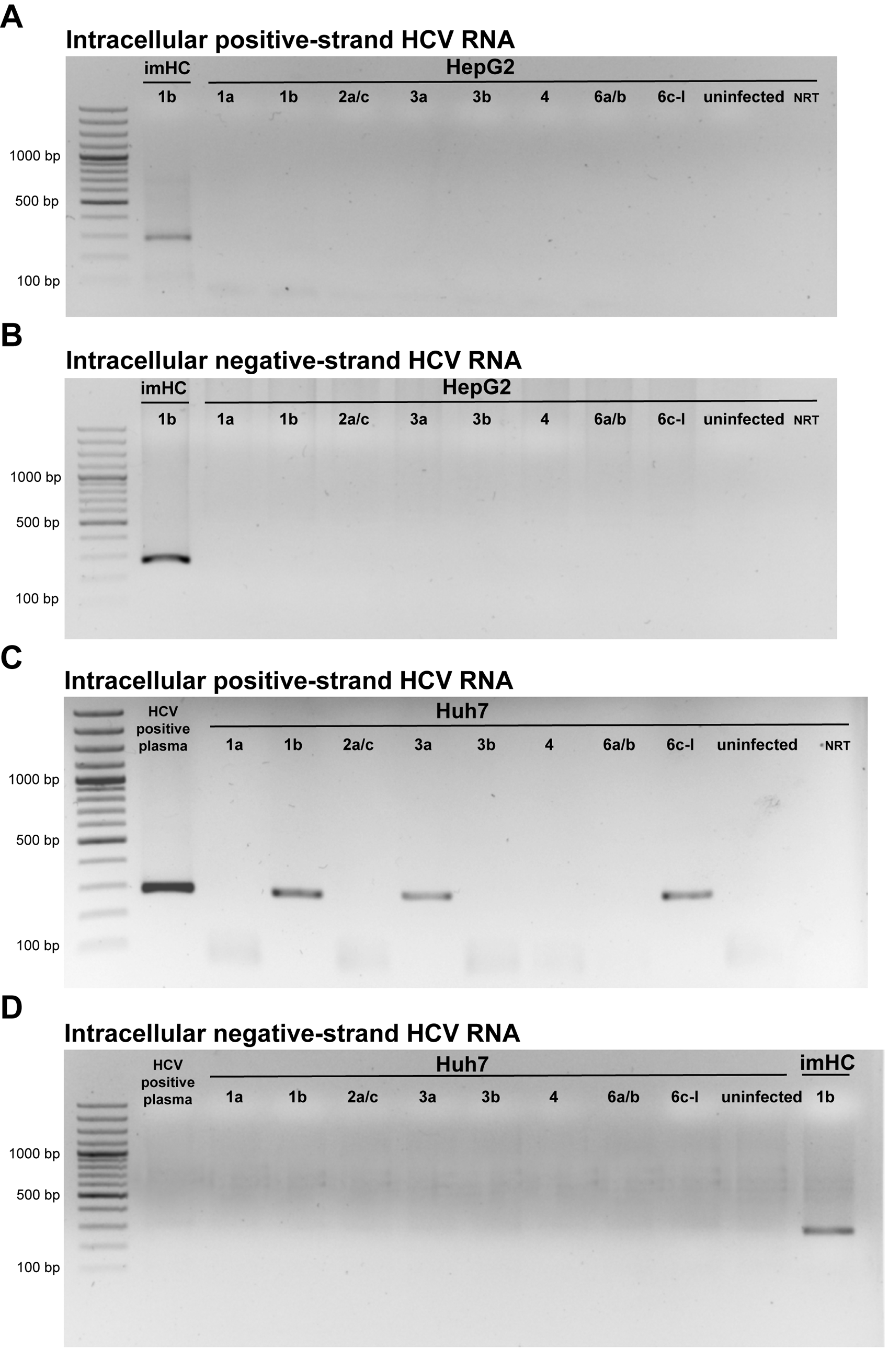

Supplement: S1 Fig — HepG2 and Huh7 were used as negative controls. HepG2 and Huh7 were infected with various HCV+-plasma genotypes (RAVL01 to RAVL08). After 24 h post-infection, hepatocytes were washed thrice with 0.1% BSA in DPBS and cultured for 7 d. The RNA was extracted from infected cell to detect HCV-positive and -negative RNA strands by RT-PCR. PCR products were visualized by gel electrophoresis. HCV-positive and -negative RNA strands were not detected in HepG2 (A, B). HCV-positive RNA strand was found in some HCV genotypes, (C), but no HCV-negative RNA strand (D), was detected in Huh7. The extracted RNA from HCV-positive plasma and HCV genotype 1b-infected imHC were used as positive controls for HCV-positive RNA strand and -negative RNA strand, respectively. (TIF) [file pone.0303265.s001.tif]

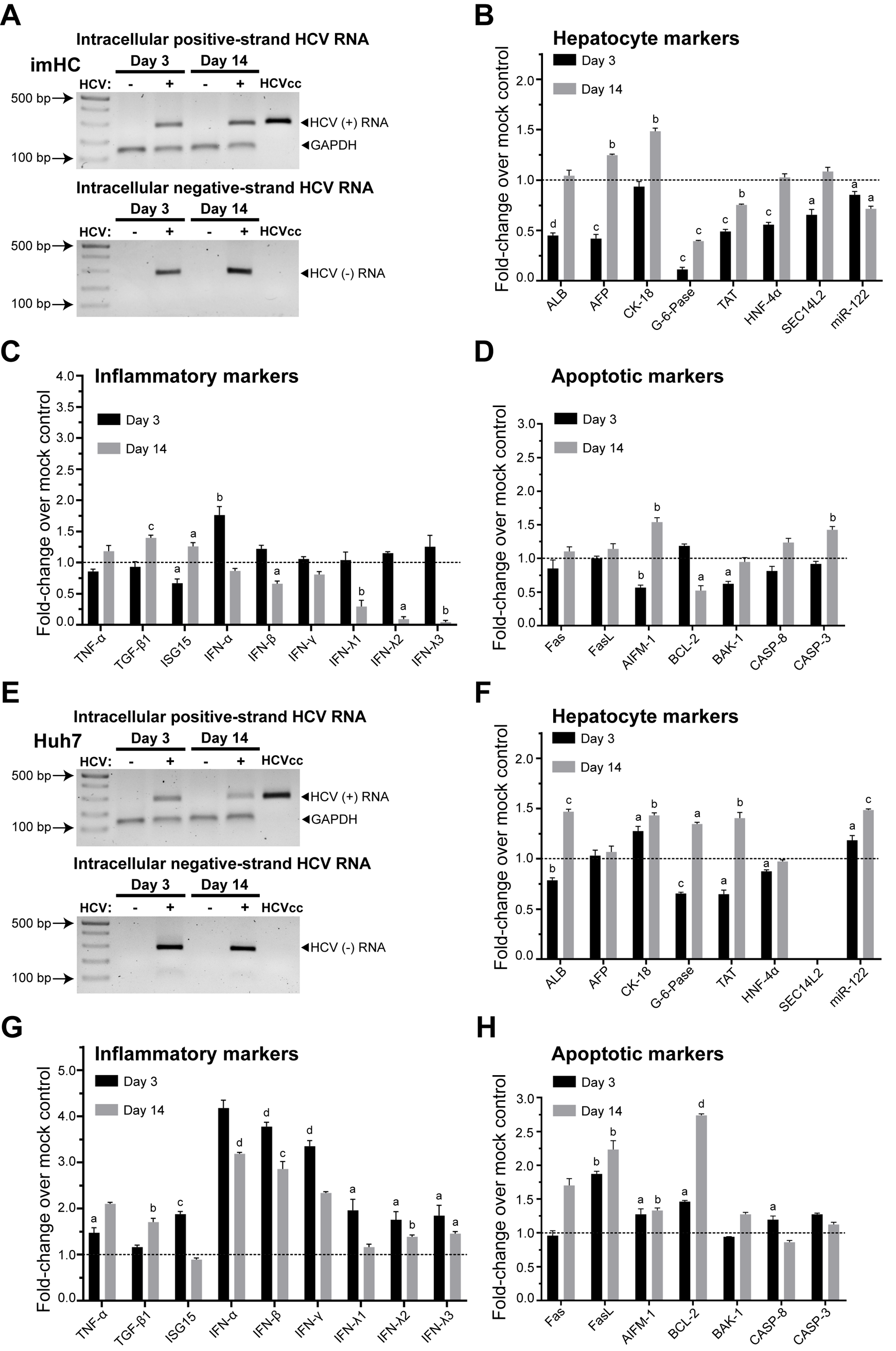

Supplement: S2 Fig — Hepatocytes were infected with HCVcc for 14 days. The intracellular HCV positive and negative RNAs were evaluated in imHC (A) and Huh7 (E) on 3 and 14 dpi. The expressions of hepatocyte markers in imHC (B) and Huh7 (F), inflammatory markers in imHC (C) and Huh7 (G), apoptotic markers in imHC (D) and Huh7 (H) were quantitated with qPCR after HCV or mock infection on 3 and 14 dpi. a, b, c, and d represented significant difference between the infection and the control with a p-value less than 0.05, 0.01, 0.001, and 0.0001 respectively. (TIF) [file pone.0303265.s002.tif]

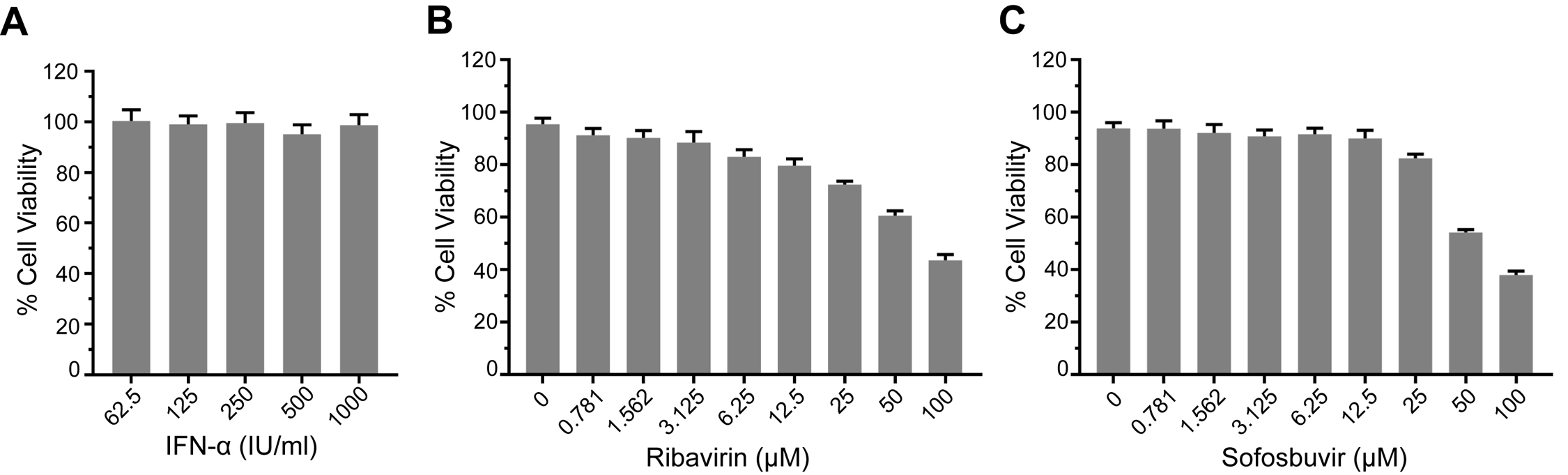

Supplement: S3 Fig — imHC (3 × 10^4^ cells per well) were incubated with IFN-α (A), ribavirin (B), or sofosbuvir (C) for 7 days. The % viability of host cells were plotted as mean ± SD from eight replicates of each concentration. The 50th percentile cytotoxic concentrations (CC50) in imHC were > 1000 IU/mL for IFN-α, 87.23 μM for ribavirin, and 66.08 μM for sofosbuvir. (TIF) [file pone.0303265.s003.tif]

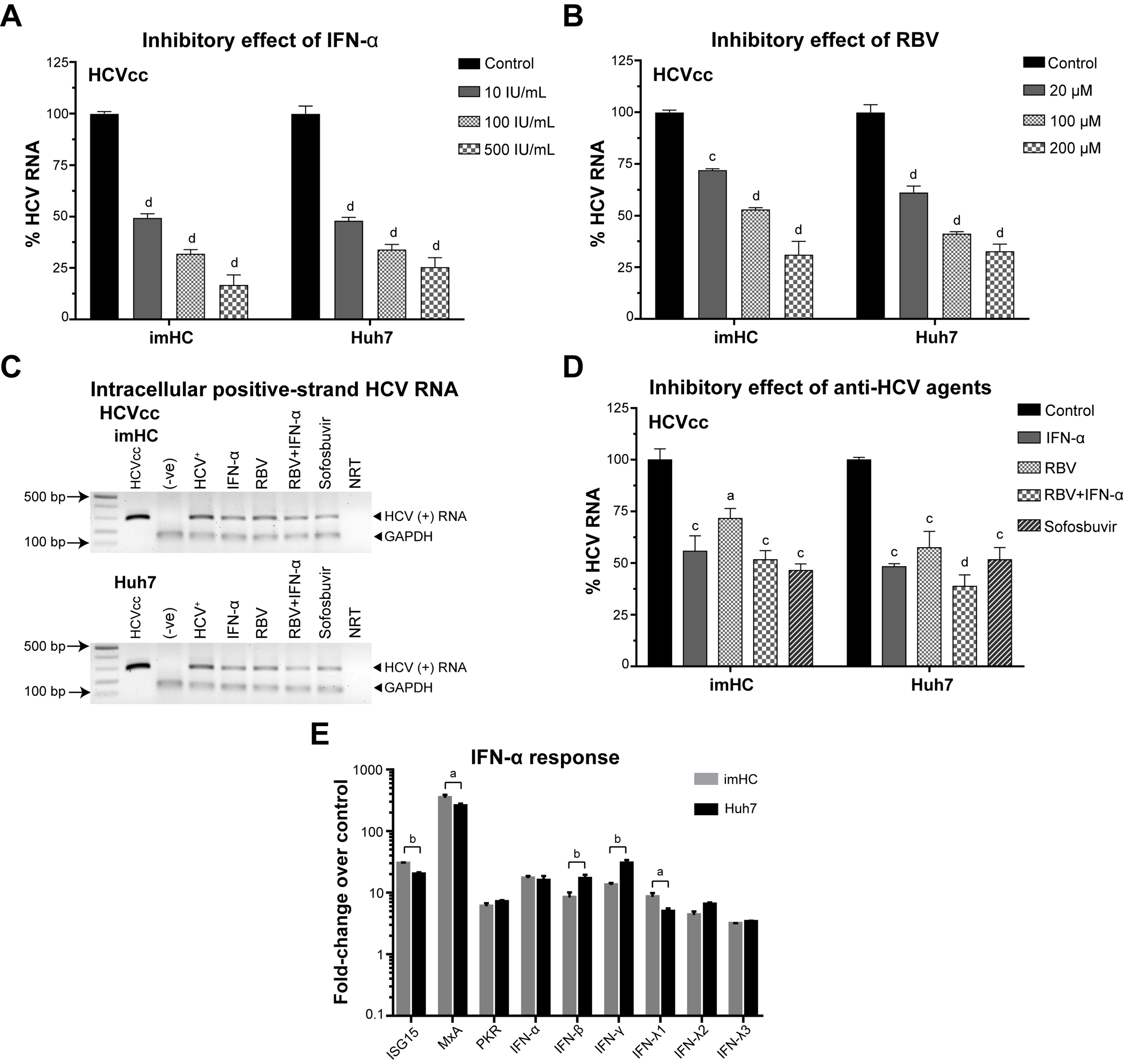

Supplement: S4 Fig — imHC and Huh7 were infected with HCVcc (genotype 2a) at MOI 1 and subsequently treated with anti-HCV agents for 7 days. The intracellular HCV positive RNA level in infected cells was determined by qPCR. The anti-HCV activity of IFN-α (A) and ribavirin (B) was evaluated. The PCR products of intracellular HCV positive RNA and GAPDH were displayed (C). The reduction of HCV RNA by IFN-α (10 IU/mL), ribavirin (20 μM), sofosbuvir (PSI-7977, 1 μM), or their combinations was investigated (D). The induction of antiviral genes response to IFN-α treatment was investigated in HCVcc-infected cells (E). These genes were plotted as fold-change over the corresponding untreated group. Abbreviations: interferon-stimulated gene 15 (ISG15), human myxovirus resistance protein 1 (MxA), protein kinase R (PKR), interferon-alpha (IFN-α), interferon-beta (IFN-β), interferon-gamma (IFN-γ), and interferon-lamda (IFN-λ). a, b, c, and d represented significant difference between cell lines or the treatments and their respective control with a p-value less than 0.05, 0.01, 0.001, and 0.0001 respectively. (TIF) [file pone.0303265.s004.tif]

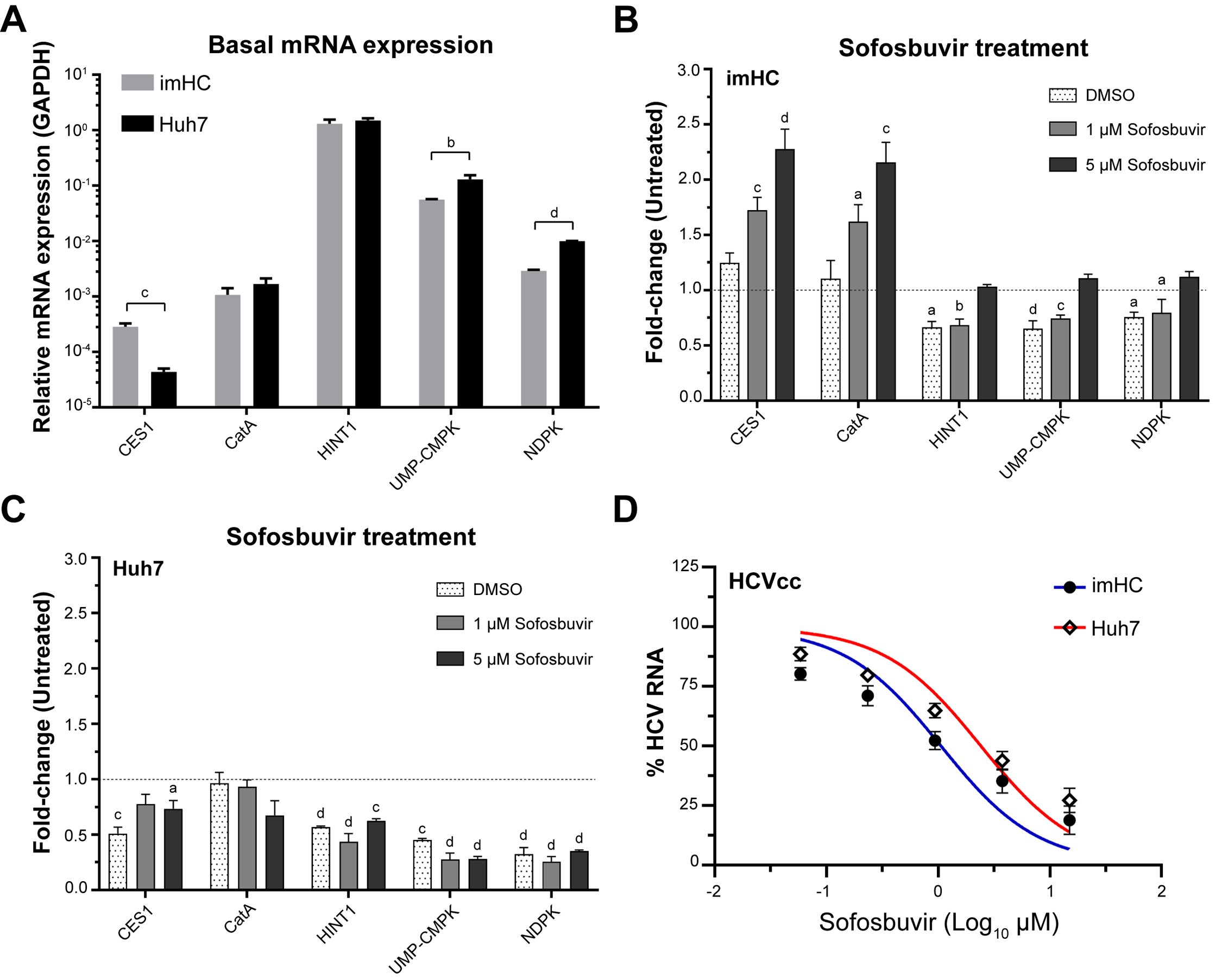

Supplement: S5 Fig — imHC and Huh7 were treated with sofosbuvir for 7 days. The expression of CES1, CatA, HINT1, UMP-CMPK, and NDPK in hepatocytes was evaluated as basal levels (A). These genes were increased in imHC (B) and were decreased in Huh7 (C) after treated with 0, 1, and 5 μM sofosbuvir. The gene expression was shown as fold-changes over the corresponding untreated groups. imHC and Huh7 were infected with HCVcc (genotype 2a) at MOI 1 and subsequently exposed to various concentrations of sofosbuvir for 7 days. The intracellular positive-stranded HCV RNA level in infected cells was evaluated as drug response compared to untreated control (D). The 50th percentile of inhibition concentrations (IC50) of sofosbuvir was 1.06 μM and 2.41 μM for imHC and Huh7 respectively. a, b, c, and d represented significant difference between cell lines or the treatments and their respective control with a p-value less than 0.05, 0.01, 0.001, and 0.0001 respectively. (TIF) [file pone.0303265.s005.tif]
