# Supplementary material for: Immortalized hepatocyte-like cells: A competent hepatocyte model for studying clinical HCV isolate infection
Source: PLoS One. 2024 May 13;19(5):e0303265. doi: 10.1371/journal.pone.0303265 (PMC11090328; doi:10.1371/journal.pone.0303265)
Supplement: S1 Table — (DOCX) [file pone.0303265.s006.docx]

| **Sample ID** | **HCV genotype** | **HCV viral load (IU/mL)** | **Experimental usage** |
| --- | --- | --- | --- |
| RAVL01 | 1a | 5,514,859 | Pan-genotype infection |
| RAVL02 | 1b | 6,997,111 | Pan-genotype infection |
| RAVL03 | 2a/c | 3,216,582 | Pan-genotype infection |
| RAVL04 | 3a | 12,691,936 | Pan-genotype infection |
| RAVL05 | 3b | 5,860,091 | Pan-genotype infection |
| RAVL06 | 4 | 1,752,337 | Pan-genotype infection |
| RAVL07 | 6a/b | 8,673,476 | Pan-genotype infection |
| RAVL08 | 6c-l | 12,691,022 | Pan-genotype infection |
| RAVL09 | 1a | 34,728,732 | IFA and Flow cytometry |
| RAVL09 | 1a | 34,728,732 | Time-course infection |
| RAVL10 | 6c-l | 21,712,214 | Re-infection |
| RAVL11 | 1b | 19,724,798 | Host-response |
| RAVL12 | 1a | 2,209,335 | Anti-HCV treatment |
| RAVL13 | 1b | 6,997,111 | Anti-HCV treatment |
| RAVL03 | 2a/c | 3,216,582 | Anti-HCV treatment |
| RAVL14 | 3a | 13,495,233 | Anti-HCV treatment |
| RAVL15 | 3b | 6,485,086 | Anti-HCV treatment |
| RAVL07 | 6a/b | 8,673,476 | Anti-HCV treatment |
| RAVL08 | 6c-l | 12,691,022 | Anti-HCV treatment |
| RAVL13 | 1b | 6,997,111 | IFN-a response |

**S1 Table.** **Data of HCV^+^-plasma samples.**
