# Supplementary material for: Immortalized hepatocyte-like cells: A competent hepatocyte model for studying clinical HCV isolate infection
Source: PLoS One. 2024 May 13;19(5):e0303265. doi: 10.1371/journal.pone.0303265 (PMC11090328; doi:10.1371/journal.pone.0303265)
Supplement: S2 Table — (DOCX) [file pone.0303265.s007.docx]

**S2 Table**. **Primer sets and conditions used in qPCR for host gene expression.**

| **Gene** | **Genbank**  **Accession** | **Sense primer**  **5’-----> 3’ (Tm °C)** | **Antisense primer**  **3’-----> 5’ (Tm °C)** | **Amplicon size (bp)** | **Annealing temp. (°C)** | **Putative function** | **References** |
| --- | --- | --- | --- | --- | --- | --- | --- |
| ALB | NM_000477.7 | TGAGAAAACGCCAGTAAGTGAC (58.87) | TGCGAAATCATCCATAACAGC (57.03) | 265 | 60 | albumin | (1) |
| AFP | NM_001134.2 | GCTTGGTGGTGGATGAAACA (58.67) | TCCTCTGTTATTTGTGGCTTTTG (57.55) | 157 | 60 | α-fetoprotein | (1) |
| CK-18 | NM_000224.3 | GAGATCGAGGCTCTCAAGGA (58.31) | CAAGCTGGCCTTCAGATTTC (57.06) | 357 | 60 | cytokeration 18 | (1) |
| G-6-Pase | NM_000151.4 | GCTGGAGTCCTGTCAGGCATTGC (65.98) | TAGAGCTGAGGCGGAATGGGAG (63.57) | 349 | 60 | Glucose-6-phosphatase | (1) |
| TAT | NM_000353.3 | TGAGCAGTCTGTCCACTGCCT (63.45) | ATGTGAATGAGGAGGATCTGAG (57.04) | 359 | 60 | tyrosine aminotransferase | (1) |
| HNF-4α | NM_178849.2 | GCCTACCTCAAAGCCATCAT (57.64) | GACCCTCCCAGCAGCATCTC (62.32) | 275 | 60 | hepatocyte nuclear factor 4α | (1) |
| NTCP | NM_003049.4 | GGACATGAACCTCAGCATTGTG (59.8) | ATCATAGATCCCCCTGGAGTAGAT (59.4) | 100 | 60 | Na+-taurocholate cotransporting polypeptide | (2) |
| MRP2 | NM_000392.5 | TGCATCTAGGCAAGGTTAACGA (59.76) | AGGAGCCATAGGTAGCCCAA (60.03) | 300 | 60 | Multidrug resistance associated protein 2 | in house design |
| CYP3A4 | NM_017460.6 | GCCTGGTGCTCCTCTATCTA (58.0) | GGCTGTTGACCATCATAAAAGC (58.23) | 187 | 60 | Cytochrome P450 (3A4) | (1) |
| Claudin-1 | NM_021101 | GTGGAGGATTTACTCCTATGCCG (59.1) | ATCAAGGCACGGGTTGCTT (59.1) | 165 | 60 | claudin-1 | (3) |
| Occludin | NM_001205255 | ACAGGCCTGATGAATTGCCA (58.5) | GTGAAGGCACGTCCTGTGT (59.3) | 218 | 60 | occludin | (3) |
| SR-B1 | NM_005505 | TGCACTATGCCCAGTACGTC (58.7) | TAGGCCTGAATGGCCTCCTT (60.3) | 148 | 60 | scavenger receptor class B type I | (3) |
| CD81 | NM_004356 | ACCTCCTGTATCTGGAGCTGG (60.0) | TTGGCGATCTGGTCCTTGTTG (59.4) | 235 | 60 | Cluster of Differentiation 81 | (3) |
| ApoE | XM_005258867 | CGCTTTTGGGATTACCTGCG (58.8) | GGGGTCAGTTGTTCCTCCAG (59.8) | 158 | 60 | Apolipoprotein E | (3) |
| ApoB | NM_000384.2 | TACCTCCGCATCTTGGGAGA (60.03) | AGGGTTTTGCCACCAGTTCA (60.03) | 307 | 60 | Apolipoprotein B | in house design |
| LDLR | NM_000527.4 | CAGTCTGGAGGATGACGTGG (59.83) | ACTGTCCGAAGCCTGTTCTG (59.97) | 177 | 60 | low density lipoprotein receptor | in house design |
| EphA2 | NM_004431.4 | CCATTAAGGACTCGGGGCAG (60.18) | TTGCCATACGGGTGTGTGAG (60.32) | 288 | 60 | Ephrin type-A receptor 2 | in house design |
| EGFR | NM_005228.5 | AACACCCTGGTCTGGAAGTACG (61.93) | TCGTTGGACAGCCTTCAAGACC (62.75) | 106 | 60 | Epidermal growth factor receptor | (4) |
| SEC14L2 | NM_012429.5 | CCAGGCAGAAGGAGGCATTG (61.04) | TCGGAGCCAACGCAGGAG (62.07) | 101 | 60 | SEC14-like protein 2 | (5) |
| TNF-α | NM_000594.3 | ATGAGCACTGAAAGCATGATCC (59.05) | GAGGGCTGATTAGAGAGAGGTC (59.11) | 217 | 60 | tumor necrosis factor-α | (6) |
| TGF-β1 | NM_000660.6 | GCGTGCTAATGGTGGAAACC (59.83) | GCTTCTCGGAGCTCTGATGTGT (62.35) | 100 | 60 | transforming growth factor beta 1 | (7) |
| ISG-15 | NM_005101.4 | CACCTGAAGCAGCAAGTGAGCGGGCTGGAG (73.7) | CCGCAGGCGCAGATTCATGAACACGGTGCT (74.1) | 150 | 60 | ISG-15 ubiquitin like modifier | (8) |
| MxA | NM_002462.5 | GCCAGCAGCTTCAGAAGGCCATGCTGCAGC (74.8) | GGGCAAGCCGGCGCCGAGCCTGCGTCAGCC (82.1) | 150 | 60 | MX dynamin like GTPase 1 | (8) |
| PKR | NM_001135651.3 | TTTGAAACATCAAAGTTTTTCACAGACCTA (60.9) | CACAGTCAAGGTCCTTAGTATTTCAGATGT (62.8) | 150 | 60 | protein kinase R | (8) |
| IFN-α | NM_024013.2 | GACTCCATCTTGGCTGTGA (56.75) | TGATTTCTGCTCTGACAACCT (57.22) | 103 | 60 | interferon alpha | (9) |
| IFN-β | NM_002176.4 | AAACTCATGAGCAGTCTGCA (57.44) | AGGAGATCTTCAGTTTCGGAGG (59.24) | 168 | 60 | interferon beta | (10) |
| IFN-γ | NM_000619.2 | GTGTGGAGACCATCAAGGAAGAC (60.87) | CAGCTTTTCGAAGTCATCTCGTTT (60.08) | 80 | 60 | interferon gamma | (7) |
| IFN-λ1 | NM_172140.1 | TGGATTGCCCATTTTGCGTG (60.04) | GAGTGACTCTTCCAAGGCGT (59.68) | 250 | 60 | interferon lambda 1 | in house design |
| IFN-λ2 | NM_172138.2 | GGTGACAGCCTCAGAGTGTTT (60.20) | AGCGACTCTTCTAAGGCATCT (58.62) | 253 | 60 | interferon lambda 2 | in house design |
| IFN-λ3 | NM_172139.4 | CATTCCCTCAGCTCCCTTTCT (59.44) | AAGCGACTCTTCTAAGGCATCT (59.24) | 228 | 60 | interferon lambda 3 | in house design |
| Fas | NM_152872.4 | TGAAGGACATGGCTTAGAAGTG (58.06) | GGTGCAAGGGTCACAGTGTT (60.75) | 118 | 60 | Fas cell surface death receptor | (11) |
| FasL | NM_000639.3 | ACACCTATGGAATTGTCCTGC (58.00) | GACCAGAGAGAGCTCAGATACG (59.45) | 311 | 60 | Fas Ligand | (11) |
| AIFM-1 | NM_004208.4 | GGGAGGACTACGGCAAAGGT (61.55) | CTTCCTTGCTATTGGCATTCG (57.66) | 101 | 60 | Apoptosis Inducing Factor Mitochondria Associated 1 | (12) |
| BCL-2 | NM_000633.3 | CTGAGTAAATCCATGCACCTAAAC (57.88) | AAACAAAACCACCAAAAGAAAGC (57.38) | 154 | 60 | BCL2 Apoptosis Regulator | in house design |
| Bak-1 | NM_001188.4 | ACGCTATGACTCAGAGTTCC (56.76) | CTTCGTACCACAAACTGGCC (59.13) | 360 | 60 | BCL2 Antagonist/Killer 1 | (13) |
| CASP-8 | NM_001228.4 | AGAGGGCTTATGATTCAGATTG (55.65) | GTGGTAGTGTGGAATTTAGCTTTG (58.30) | 100 | 60 | Caspase 8 | in house design |
| CASP-3 | NM_004346.4 | ATGAGCACATAGGACTCTA (51.80) | AAGAAATCTCCCGTGAAAT (51.72) | 150 | 60 | Caspase 3 | (12) |
| CES1 | NM_001025194.2 | CACCCAAGATCCCAAGGCG (60.75) | CACCACGTTTTCATGGGCAG (60.04) | 232 | 60 | carboxylesterase 1 | PrimerBank (ID 68508966c1) |
| CatA | NM_000308.4 | AGAGCAAGGACGCGGG (58.93) | TTCTCGGGATCCTTCTGGGA (59.66) | 249 | 60 | cathepsin A | in house design |
| HINT1 | NM_005340.7 | GATCATCCGCAAGGAAATACCA (58.52) | TCACCACCATTCGATAACCCT (58.81) | 233 | 60 | histidine triad nucleotide binding protein 1 | PrimerBank (ID 218777823c1) |
| UMP-CMPK | NM_016308.3 | TCCTCTGCTCTCCACGTCTC (60.68) | GCAGAAAGGTGTGTGTAGCC (59.12) | 121 | 60 | cytidine/uridine monophosphate kinase 1 | in house design |
| NDPK | NM_198175.1 | AGGACTAAGTCAGCCTGGTG (58.73) | CGCCTTGAAAGACGATCCCT (60.11) | 156 | 60 | NME/NM23 nucleoside diphosphate kinase 1 | in house design |
| GAPDH | NM_002046.7 | GAAATCCCATCACCATCTTCC (55.0) | AAATGAGCCCCAGCCTTCTC (59.6) | 124 | 60 | Glyceraldehyde-3-Phosphate Dehydrogenase | (3) |
| miR-122 RT (RT primer) | - | GTCGTATCCAGTGCGTGTCGTGGAGTCGGCAATTGCACTGGATACGACCAAACAC | - | - | - | - | (14) |
| miR-122 | NR_029667.1 | GGGGTGGAGTGTGACAATG (58.05) | CAGTGCGTGTCGTGGAGT (59.97) | 85 | 60 | MiroRNA 122 | (14) |
| U6 RT (RT primer) | - | CGCTTCACGAATTTGCGTGTCAT | - | - |  | - | (15) |
| U6 | NR_104084.1 | GCTTCGGCAGCACATATACTAAAAT (60.05) | CGCTTCACGAATTTGCGTGTCAT (63.00) | 89 | 60 | U6 small nuclear RNA | (15) |

**References**

1. Sa-ngiamsuntorn K, Wongkajornsilp A, Kasetsinsombat K, Duangsa-ard S, Nuntakarn L, Borwornpinyo S, et al. Upregulation of CYP 450s expression of immortalized hepatocyte-like cells derived from mesenchymal stem cells by enzyme inducers. BMC Biotechnol. 2011;11(1):89.

2. Thongsri P, Pewkliang Y, Borwornpinyo S, Wongkajornsilp A, Hongeng S, Sa-Ngiamsuntorn K. Curcumin inhibited hepatitis B viral entry through NTCP binding. Sci Rep. 2021;11(1):19125.

3. Sa-Ngiamsuntorn K, Wongkajornsilp A, Phanthong P, Borwornpinyo S, Kitiyanant N, Chantratita W, et al. A robust model of natural hepatitis C infection using hepatocyte-like cells derived from human induced pluripotent stem cells as a long-term host. Virol J. 2016;13(1):59.

4. Jia X, Mo Z, Zhao Q, Bao T, Xu W, Gao Z, et al. Transcriptome alterations in HepG2 cells induced by shRNA knockdown and overexpression of TMEM2 gene. Biosci Biotechnol Biochem. 2020;84(8):1576-84.

5. Ni J, Wen X, Yao J, Chang HC, Yin Y, Zhang M, et al. Tocopherol-associated protein suppresses prostate cancer cell growth by inhibition of the phosphoinositide 3-kinase pathway. Cancer Res. 2005;65(21):9807-16.

6. Rajput S, Volk-Draper LD, Ran S. TLR4 is a novel determinant of the response to paclitaxel in breast cancer. Mol Cancer Ther. 2013;12(8):1676-87.

7. Wongkajornsilp A, Wamanuttajinda V, Kasetsinsombat K, Duangsa-ard S, Sa-ngiamsuntorn K, Hongeng S, et al. Sunitinib indirectly enhanced anti-tumor cytotoxicity of cytokine-induced killer cells and CD3(+)CD56(+) subset through the co-culturing dendritic cells. PLoS One. 2013;8(11):e78980.

8. Kaneko S, Kakinuma S, Asahina Y, Kamiya A, Miyoshi M, Tsunoda T, et al. Human induced pluripotent stem cell-derived hepatic cell lines as a new model for host interaction with hepatitis B virus. Sci Rep. 2016;6:29358.

9. Colantonio AD, Epeldegui M, Jesiak M, Jachimowski L, Blom B, Uittenbogaart CH. IFN-alpha is constitutively expressed in the human thymus, but not in peripheral lymphoid organs. PLoS One. 2011;6(8):e24252.

10. Jaworska J, Gravel A, Fink K, Grandvaux N, Flamand L. Inhibition of transcription of the beta interferon gene by the human herpesvirus 6 immediate-early 1 protein. J Virol. 2007;81(11):5737-48.

11. Das H, Koizumi T, Sugimoto T, Chakraborty S, Ichimura T, Hasegawa K, et al. Quantitation of Fas and Fas ligand gene expression in human ovarian, cervical and endometrial carcinomas using real-time quantitative RT-PCR. Br J Cancer. 2000;82(10):1682-8.

12. Phanthong P, Borwornpinyo S, Kitiyanant N, Jearawiriyapaisarn N, Nuntakarn L, Saetan J, et al. Enhancement of beta-Globin Gene Expression in Thalassemic IVS2-654 Induced Pluripotent Stem Cell-Derived Erythroid Cells by Modified U7 snRNA. Stem Cells Transl Med. 2017;6(4):1059-69.

13. Kholoussi NM, El-Nabi SE, Esmaiel NN, Abd El-Bary NM, El-Kased AF. Evaluation of Bax and Bak gene mutations and expression in breast cancer. Biomed Res Int. 2014;2014:249372.

14. Wu X, Wu S, Tong L, Luan T, Lin L, Lu S, et al. miR-122 affects the viability and apoptosis of hepatocellular carcinoma cells. Scandinavian journal of gastroenterology. 2009;44(11):1332-9.

15. Yao R, Ma Y, Du Y, Liao M, Li H, Liang W, et al. The altered expression of inflammation-related microRNAs with microRNA-155 expression correlates with Th17 differentiation in patients with acute coronary syndrome. Cell Mol Immunol. 2011;8(6):486-95.
