# Supplementary material for: Immortalized hepatocyte-like cells: A competent hepatocyte model for studying clinical HCV isolate infection
Source: PLoS One. 2024 May 13;19(5):e0303265. doi: 10.1371/journal.pone.0303265 (PMC11090328; doi:10.1371/journal.pone.0303265)
Supplement: S3 Table — (DOCX) [file pone.0303265.s008.docx]

**S3 Table**. **Primer sets and conditions used to detect HCV RNA.**

| **Detection** | **Sequences of primers** | **Amplicon size (bp)** | **Conditions** | **References** |
| --- | --- | --- | --- | --- |
| **Positive-strand HCV RNA**  Intracellular HCV (+) RNA  HCV viral load | 5′-CCCTGTGAGGAACTACTGTCTTCACGCA-3′  5′-ACTCGCAAGCACCCTATCAGG-CAGTAC-3′  5′-TCTGCGGAACCGGTGAGTA-3′  5′-TCAGGCAGTACCACAAGGC -3′ | 270  150 | 2 min at 50°C, 10 min at 95°C; 40 cycles of 95°C for 30 s, 59°C for 1 min, and 72°C for 90 s.  2 min at 50°C, 10 min at 95°C; 40 cycles of 95°C for 30 s, 59°C for 1 min, and 72°C for 90 s. | (1)  (2) |
| **Negative-strand HCV RNA**  Reverse transcription  I PCR  II PCR | 5′-TGRTGCACGGTCTACGAGACCTC-3′  5′-RAYCACTCCCCTGTGAGGAAC-3′    5′-ACTGTCTTCACGCAGAAAGCGTC-3′  5′-CAAGCACCCTATCAGGCAGTACC-3′ | -  -  251 | Following the protocol  (ImProm-II™, Promega, WI)  3 min at 95°C; 50 cycles of 95°C for 15 s, 58°C for 30 s, and 72°C for 30 s.  3 min at 95°C; 30 cycles of 95°C for 15 s, 58°C for 30 s, and 72°C for 30 s. | (3) |

**References**

1. Raymond VA, Selliah S, Ethier C, Houle R, Jouan L, Maniere T, et al. Primary cultures of human hepatocytes isolated from hepatitis C virus-infected cirrhotic livers as a model to study hepatitis C infection. Liver international : official journal of the International Association for the Study of the Liver. 2009;29(6):942-9.

2. Zhao H, Lin W, Kumthip K, Cheng D, Fusco DN, Hofmann O, et al. A functional genomic screen reveals novel host genes that mediate interferon-alpha's effects against hepatitis C virus. J Hepatol. 2012;56(2):326-33.

3. Pawelczyk A, Kubisa N, Jablonska J, Bukowska-Osko I, Caraballo Cortes K, Fic M, et al. Detection of hepatitis C virus (HCV) negative strand RNA and NS3 protein in peripheral blood mononuclear cells (PBMC): CD3+, CD14+ and CD19+. Virol J. 2013;10(1):346.
